# Physiological and Transcriptome Indicators of Salt Tolerance in Wild and Cultivated Barley

**DOI:** 10.3389/fpls.2022.819282

**Published:** 2022-04-14

**Authors:** Narges Gharaghanipor, Ahmad Arzani, Mehdi Rahimmalek, Rudabeh Ravash

**Affiliations:** ^1^Department of Agronomy and Plant Breeding, College of Agriculture, Isfahan University of Technology, Isfahan, Iran; ^2^Department of Horticulture, College of Agriculture, Isfahan University of Technology, Isfahan, Iran; ^3^Department of Plant Breeding and Biotechnology, Faculty of Agriculture, Shahrekord University, Shahrekord, Iran

**Keywords:** functional genomics, isoform, mRNA transcript, pathway, physiology, salinity

## Abstract

Barley is used as a model cereal to decipher salt tolerance mechanisms due to its simpler genome than wheat and enhanced salt tolerance compared to rice and wheat. In the present study, RNA-Seq based transcriptomic profiles were compared between salt-tolerant wild (*Hordeum spontaneum*, genotype no. 395) genotype and salt-sensitive cultivated (*H. vulgare*, ‘Mona’ cultivar) subjected to salt stress (300 mM NaCl) and control (0 mM NaCl) conditions. Plant growth and physiological attributes were also evaluated in a separate experiment as a comparison. Wild barley was significantly less impacted by salt stress than cultivated barley in growth and physiology and hence was more stress-responsive functionally. A total of 6,048 differentially expressed genes (DEGs) including 3,025 up-regulated and 3,023 down-regulated DEGs were detected in the wild genotype in salt stress conditions. The transcripts of salt-stress-related genes were profoundly lower in the salt-sensitive than the tolerant barley having a total of 2,610 DEGs (580 up- and 2,030 down-regulated). GO enrichment analysis showed that the DEGs were mainly enriched in biological processes associated with stress defenses (e.g., cellular component, signaling network, ion transporter, regulatory proteins, reactive oxygen species (ROS) scavenging, hormone biosynthesis, osmotic homeostasis). Comparison of the candidate genes in the two genotypes showed that the tolerant genotype contains higher functional and effective salt-tolerance related genes with a higher level of transcripts than the sensitive one. In conclusion, the tolerant genotype consistently exhibited better tolerance to salt stress in physiological and functional attributes than did the sensitive one. These differences provide a comprehensive understanding of the evolved salt-tolerance mechanism in wild barley. The shared mechanisms between these two sub-species revealed at each functional level will provide more reliable insights into the basic mechanisms of salt tolerance in barley species.

## Introduction

Soil salinization is a serious threat to agricultural lands in arid and semi-arid regions. Barley (*Hordeum vulgare* ssp. *vulgare*; hereafter referred to as *H. vulgare*) is an important salt-tolerant cereal crop and is among the world′s earliest domesticated crop plants. *H. vulgare* ssp. *spontaneum* (hereafter referred to as *H. spontaneum*) is a wild progenitor of barley and grows over an astonishingly wide range of ecological habitats, including highlands and deserts (Jakob et al., 2014). There is substantial genetic variation in the barley gene pools for salt tolerance to ensure the continued improvement of barley adaptation to salt stress (Garthwaite et al., 2005; Ebrahim et al., 2020). However, the process of domestication, subsequent improvement, and cultivation in less stressful environments may lead to loss of genetic variation and evolutionary adaptation potential in cultivated barley (Ellis et al., 2000).

A comprehensive understanding of molecular mechanisms underlying salt tolerance in crop plants would be highly desirable for the development of salt-tolerant cultivars through cloning of candidate genes and genetic engineering (Deinlein et al., 2014). “Salt tolerance” is defined as the ability to limit the detrimental effects of salt by a plant genotype through alleviating osmotic stress, ion toxicity, and the resultant oxidative stress, thereby diminishing yield loss (Munns and Tester, 2008; Arzani and Ashraf, 2016). At the molecular level, a large number of genes are associated with salt tolerance and are mainly involved in signaling, phytohormone biosynthesis, ion transporter and channels, transcription factors, and reactive oxygen species (ROS) scavenging (Formentin et al., 2018; Fu et al., 2018).

Transcriptome analysis is a powerful tool to decipher the physiological, biochemical, and molecular adaptations related to plant response to stresses. This method allows predicting of genes and their biological functions involved in stress tolerance mechanisms and, hence, to connect the genotype to the phenotype of a cell. RNA sequencing (RNA-Seq) technologies coupled with existing reference genome and bioinformatic analysis can provide an unbiased profile of the plant transcriptome. To aid in this endeavor, contrasting (tolerant and sensitive) genotypes can be employed to decipher the comparative transcriptomic alterations at normal and salt stress conditions (Wu et al., 2018).

Over the past decade, there have been many studies that have implemented RNA-Seq technology to comparatively analyze the transcriptome changes induced by salt stress of the tolerant and sensitive genotypes in oat (Wu et al., 2018), rice (Mansuri et al., 2019), soybean (Zeng et al., 2019), and bermudagrass (Hu et al., 2015). An RNA-Seq differential expression analysis can be used to identify salt tolerance genes in a salt-tolerant wild ancestor that may provide a better understanding of underlying mechanisms and pathways and subsequently incorporate desired genes into cultivated species. There is only one study, to our knowledge, that has investigated the RNA-Seq based transcriptome analysis of a single genotype of *H. spontaneum*. As such, the objective of this study is to employ a comparative RNA-Seq study to elucidate the molecular mechanisms underlying the adaptive strategies used by wild barley genotype to combat salt stress when compared with the salt-sensitive *H. vulgare* cultivar. Physiological and functional outcomes were assessed as a function of salt stress and several mechanisms were advanced to explain the observed effects in contrasting barley genotypes.

## Materials and Methods

### Plant Materials and Growing Conditions

Two barley genotypes belonging to the wild subspecies *H. spontaneum* (a salt-tolerant genotype, no. 395) and a salt-sensitive cultivar (‘Mona’) were used in this study. This genotype was selected from the 47 *H. spontaneum* genotypes collected from West Iran (see Ebrahim et al., 2020). The individual plant was taken from each collection site. The collected seeds from each plant (pure line; hereafter termed “genotype”) were subsequently planted in separate rows to increase seeds in the field conditions. Seed germination was performed on moist filter paper at 4°C. The seedlings were moved to plastic pots with sand culture medium (fine size 50–200 micrometers) in the greenhouse conditions at a photoperiod of 12L:12D with a photosynthetic photon flux density (PPFD) of ∼400 μmol m^–2^ s^–1^, day–night temperatures 25 ± 3°C–17 ± 3°C, and the relative humidity ∼50%. Plants were irrigated every 2 days and fertilized once a week with half-strength Hoagland’s solution (Hoagland and Arnon, 1950). At the two-leaf stage, half of the pots were watered normally (control) and the other half were subjected to salt stress for 6 days at 300 mM NaCl in the two experiments of this study.

For the RNA-Seq analysis, the fresh leaves were collected from 10 plants in each entry, immediately frozen in liquid nitrogen, and kept at −80°C until the extraction of RNA. Two biological replicates per treatment (control and salt stress) and per genotype (tolerant and sensitive) were used in the RNA-Seq study.

### Physiological Study

A 2 × 2 factorial experiment that was spread across four replicate blocks was used. Plant height was measured to the tip of the uppermost leaf. Leaf and root dry weight was obtained after drying the plant tissues in an oven at 70°C for 72 h. Leaf membrane stability index (MSI) was measured in control and salt-treated plants according to Premachandra et al. (1990). To evaluate the lipid peroxide content of the leaves, the malondialdehyde (MDA) content was assessed using the thiobarbituric acid (TBA) reaction method reported by Taulavuori et al. (2001). The relative water content (RWC) (Ebrahim et al., 2020) and stress tolerance index (STI) (Fernandez, 1992) were calculated. In order to measure the concentrations of sodium (Na), potassium (K) ions of the root and shoot tissues, the samples were dried at 70°C for 48 h and were then incinerated at 550°C for 4 h. The acid digested (10 ml 2N HCl) samples were then analyzed for measuring the concentration of Na and K using a flame emission photometry (Jenway PF P7 Flame Photometer; Jenway, Essex, United Kingdom). A standard curve was used to calculate the Na and K concentrations (Houshmand et al., 2005).

The statistical significance of environment and genotype and their interaction was determined using factorial analysis of variance (ANOVA). The data were analyzed using the GLM procedure of SAS software (version 9.4; SAS Institute Inc., Cary, NC, USA). The means of variables were compared using Tukey’s HSD test.

### RNA Sequencing

Total RNA was extracted using RNA Isolation Kit (Roche, Germany) as per the guidance of the manufacturer’s protocol. The quality and quantity of total RNA quality were checked by a NanoDrop 8000 spectrophotometer. Isolated RNA was treated with DNaseI to remove contaminating DNA. Total RNA samples were subsequently sent for transcriptome analysis at Macrogen Co., Seoul, South Korea. RNA-Seq libraries were constructed using the TruSeq Stranded mRNA LT Sample Prep Kit (Illumina) following the manufacturer’s instruction guide (Illumina Part # 15031047 Rev. E). Illumina HiSeq 2500 platform was used to sequence the libraries, using 151 bp paired-end reads. Cutadapt (Martin, 2011) or Trimmomatic (v.0.39; Bolger et al., 2014) was used to remove the reads containing adaptor contamination, undetermined bases, and low-quality bases. The assessment of the quality of the sequencing data was carried out using FastQC^1^, before further bioinformatic analysis. FastQC reports the number of reads, read length, K-mer content, presence of the ambiguous base, and duplicated reads.

### Differential Expression Genes and Differential Expression Isoforms

Four groups of the RNA-Seq data: tolerant – control (TC), tolerant – stress (TS), sensitive – control (SC), sensitive – stress (SS) each with two biological replications were subjected to the analyses described below. RNA sequencing resulted in an average per sample of total reads of 7.8 Gb and paired-end reads of 50 million. A total of 453 million clean reads with a range of 40 to 66 million read pairs were obtained from the paired-end reads of the eight samples. Fast QC report the number of reads, read length, K-mer content, presence of the ambiguous base, duplicated. Initially, the raw read was processed to remove the adaptor and filter low-quality (Phred score ≥ 30) and short reads (shorter than 60 bp) using Trimmomatic software v0.39 (Bolger et al., 2014). First, the clean reads from each dataset (eight samples) were aligned to the barley cultivar ‘Morex’ reference genome (Ensemble database; Hordeum_vulgare.IBSC_v2.dna.toplevel.fa) using STAR (Dobin et al., 2013). Then, the expression of all genes and isoforms was determined using RSEMv1.3.2 (Li and Dewey, 2011). Generated reads were mapped to the barley genome using gSNAP. Then Cufflinks v. 2.2.1 was adapted to estimate the expression values as fragments per kilobase of transcript per million mapped reads (FPKM). Statistical analysis for RNA-seq was performed using the DESeq2 package in R (Love et al., 2014). Genes and isoforms with the FDR adjusted *p* value < 0.05 and | log2 fold change (FC)| ≥ 1.5 were considered as differentially expressed (DE). The expression changes between the four contrast groups of experimental treatments viz. tolerant genotype grown in control (TC) vs. grown in salt stress (TS), sensitive genotype grown in control (SC) vs. grown in salt stress (SS), tolerant vs. sensitive genotype grown in control (TC-SC), tolerant vs. sensitive genotype grown in salt stress (TS-SS) conditions were assessed. The frequency distribution of the number of isoforms per gene and their abundance in two *H. vulgare* subspecies (salt-tolerant and sensitive) was counted. To visualize the common and the unique differentially expressed genes (DEGs) in the four contrasting groups (TC, TS, SC, and SS), the Venn diagram software^2^ was used. Group comparisons were performed using the Chi-square (χ^2^) and Mann–Whitney *U* tests, as appropriate.

### Functional Annotation and Gene Ontology Enrichment Analysis

Gene Ontology (GO) enrichment analysis was undertaken to explore the function of the DEGs associated with salt stress. Classification of DEGs to GO terms was done using the g: Profiler^3^ (Raudvere et al., 2019). The GO was used to assess the enrichment of various categories of GO for the genes with |log2 FC| ≥ 1.5 in the wild and cultivated barleys. g:Profiler uses cumulative hypergeometric P-values to identify the most significant GO terms corresponding to the input set of genes. PANTHER^4^ was first adopted to understand gene ontology and function of the DEGs. To further examine the significance of the overrepresented (enriched) GO terms within GO categories of molecular function and biological process, the Functional Annotation Chart tool of the Database for Annotation, Visualization and Integrated Discovery (DAVID)^5^ was applied with a modified non-parametric Fisher’s exact test to identify. Then, based on the generated *p* value, a particular canonical pathway was assigned to the DEGs by Kyoto Encyclopedia of Genes and Genomes (KEGG)^6^. Finally, putative protein kinases (PKs), transcription factors (TFs), and transcriptional regulators (TRs) were identified by a homologous search of the un-assigned DEGs against the iTAK database using the BlastX (Zheng et al., 2016).

### Expression Validation Using Quantitative Real-Time PCR

Seven salt-responsive genes (DEGs) were randomly selected to validate the transcriptome RNA-Seq data. These include five upregulated [potassium transporter (*HvKT24*), chloride channel protein (*HvCLC-c*), ABA-responsive element binding factor (ABF, *HvbZIPx*), dehydrin 7 (*AvDhn7*), heat shock protein (HSP, *HvHSP20*)] and two down-regulated (calcium-sensing receptor and cytochrome c) genes. Targeted seven pairs of primers listed in Supplementary Table 1 were designed using Primer 3 software^7^. The control and salt-stressed plants of the two-barley subspecies were grown and treated using a similar protocol to that employed for RNA-Seq analysis. In addition, the original samples used for RNA sequencing analysis were also utilized for quantitative real-time PCR (qRT-PCR) analysis. Two biological replicates and two technical replicates were used for each qRT-PCR reaction. Total RNA of the leaf tissues was extracted using a Plant RNA Isolation Kit (Qiagen, Hilden, Germany) following the manufacturer’s instruction. The RNA samples were treated with DNase I, quality checked by a Nanodrop spectrophotometer, and reverse transcribed into complementary DNA (cDNA). qRT-PCR was used to assess the expression of seven candidate genes using Real Q Plus 2x Master Mix Green (Ampliqon, Denmark) on the ABI StepOnePlus Real-Time PCR System (Applied Biosystems). Fold change in relative mRNA expression of the target genes was calculated by the 2^–^ΔΔCt method (Livak and Schmittgen, 2001). The threshold cycle for different samples was normalized using the β*-tubulin* reference gene. The Mann–Whitney *U* test was used to determine whether there were significant differences in transcript expression between salt-tolerant and salt-sensitive genotypes relative to the control.

## Results

### Physiological Study

Table 1 shows the means of growth and physiological parameters evaluated in two contrasting genotypes of barleys (salt-tolerant and sensitive) grown in control and salt stress treatments. In response to salt stress, root and leaf Na concentrations, MSI, and MDA were increased significantly. In contrast, the root and leaf K concentrations, K/Na ratio, leaf RWC, plant height, root and shoot DW were significantly decreased due to salt stress. However, wild barley had significantly better root and shoot growth and was less affected by salt stress for the physiological attributes studied (Table 1). The two barleys significantly differed for the element concentrations of the above ground parts and the roots under both control and salt-stress conditions. The concentration of MDA was increased in response to salt stress in both the wild genotype and Mona barley, but the increase was more pronounced in the case of the ‘Mona’ cultivar. In response to salt stress, the RWC showed a significantly higher reduction in the Mona cultivar than the wild genotype. Tolerant wild genotype had lower cell electrolyte leakage that indicated a greater MSI than sensitive plants when exposed to 300 mM NaCl. The calculated STI was higher for the tolerant genotype (0.71) compared to that in the sensitive genotype (0.21).

**TABLE 1:** Mean comparisons of tolerant and sensitive barleys for growth and physiological traits in control and salt stress conditions.

Trait	Tolerant		Sensitive	
	Control	Salt stress	Control	Salt stress
Shoot dry weight (g)	0.57^a^*	0.37^b^	0.47^a^	0.18^c^
Root dry weight (g)	0.91^a^	0.11^c^	0.55^b^	0.08^c^
Plant height (cm)	53^a^	43^b^	49.5^a^	34.33^c^
MSI (%)	98.02^a^	75.8^b^	92.55^ab^	22.65^c^
RWC (%)	95.62^a^	72.42^b^	89.88^a^	43.91^c^
Leaf Na concentration (mmol/g DW)	0.62^c^	1.78^b^	0.64^c^	4.57^a^
Leaf K concentration (mmol/g DW)	1.01^a^	0.73^b^	1.046^a^	0.45^b^
Leaf K/Na ratio	1.62^a^	0.42^b^	1.71^a^	0.10^c^
Root Na concentration (mmol/g DW)	1.081^c^	1.49^b^	1.06^c^	2.7^a^
Root K concentration (mmol/g DW)	0.62^a^	0.36^ab^	0.26^ab^	0.15^c^
Root K/Na ratio	0.64^a^	0.25^ab^	0.28^ab^	0.05^c^
Leaf MDA content (nmol/g FW)	4.59^b^	6.43^b^	2.13^c^	11.80^a^

*Means with the same letter in each row are not significantly different using Tukey’s HSD test at p < 0.05.

*MSI, membrane stability index; RWC, relative water content.*

**FIGURE 1:**
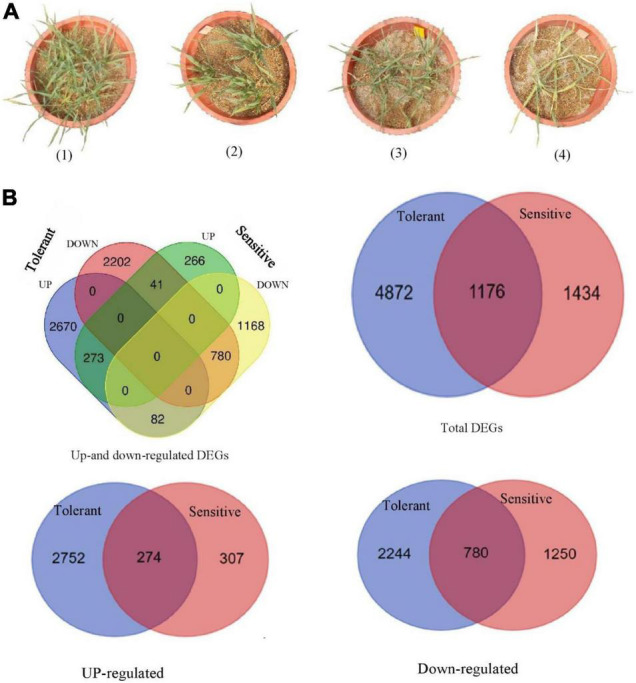
**(A)** Growth performance of tolerant genotype (pot no. 1 and 3) and sensitive cultivar (pot no. 2 and 4) under control (two pots on the left) and salt stress (two pots on the right) conditions. **(B)** Venn diagrams showing the number of distinct and common salt-responsive genes (DEGs) found in two barley subspecies (wild salt-tolerant genotype and ‘Mona’ salt-sensitive cultivar).

Plant height was reduced by salt stress by 19% and 31%, in the salt-tolerant and salt-sensitive genotypes, respectively. The DW of the shoots and roots were significantly decreased in response to salt stress. Figure 1A shows the growth performance of the two barley genotypes grown in salt stress and control conditions. The Na concentrations of the roots and leaves were much more prominently accumulated in the sensitive than tolerant genotype. In contrast, the K concentrations of the roots and leaves were diminished in the two barleys at 300 mM NaCl, but they were even sharper in the ‘Mona’ cultivar. The K/Na ratio, as a salt stress indicator in cereals, declined significantly under salt stress in two barleys, but the decrease was much lower in wild than cultivated barley.

### Transcriptome Analysis

#### Overview of the RNA-Seq

Eight libraries, obtained from the transcriptome profiles of leaf tissues of control and salt-treated plants in two barleys, were subjected to a deep RNA sequencing (RNA-Seq) analysis. The cleaned and trimmed reads of each sample were mapped to the reference barley genome sequence. A higher mapping rate with 77–89% reads was obtained when the data of wild genotype (tolerant) were mapped to the reference genome, than when the ‘Mona’ cultivar (sensitive) was mapped (71–80% reads). An overall RNA-Seq data analysis pipeline revealed that the two environments (saline and control) and two genotypes were distinct from each other. Four contrast groups (TC vs. TS, SC vs. SS, TC vs. SC, and TS vs. SS) were subjected to analysis of differential gene expression to compare the differences in gene expression between wild genotype and ‘Mona’ cv, and between control and salt-stress treatments in each of the genotypes.

#### Differential-Expressed Genes and Isoforms

Enrichment analysis was performed to categorize DEGs of the barley subspecies (wild and cultivated) enriched in response to salt stress. In wild barley, 6,048 DEGs were found between salt and control conditions, and of these, 3,025 genes were up-regulated and 3,023 genes were down-regulated. In the salt-sensitive cultivar (‘Mona’) 2610 DEGs including 580 up-regulated and 2,030 down-regulated genes were identified. The number of DEGs found in wild barley was 2.3 folds greater than the DEGs found in sensitive barley cultivar. Figure 1B shows the Venn diagram generated to illustrate the similarities and differences of the transcriptome changes associated with salt stress in the two contrasting genotypes. A total of 1,176 overlapping (common) DEGs were identified between the two genotypes, of which 273 and 780 genes showed a consistent pattern of up-and down-regulation, respectively. On the other hand, the expression of 4,872 DEGs was specifically induced in the tolerant genotype, and 1,434 DEGs were uniquely induced in the sensitive one. A frequency histogram of the DEGs based on the fold change in expression (Log FC) is shown in Supplementary Figure 1. The DEGs were discretized into seven groups in both control and salt stress conditions, and group one contained most of the DEGs with a fold change of 2 or 3, whereas group 7 had the least number of DEGs (fold change >8). The distribution of the assigned DEGs among the seven barley chromosomes is shown in Table 2. They were fairly evenly distributed among the seven chromosomes, but 4H had a greater number of the DEGs. In addition, two and zero up-regulated DEGs, as well as 6 and 12 down-regulated ones, were assigned to the mitochondrion in tolerant and sensitive genotypes, respectively. Twenty-five and 29 down-regulated DEGs were assigned to the plastids in tolerant and sensitive genotypes, respectively, although no up-regulated could be assigned to either cultivar.

**TABLE 2:** The differentially expressed genes (DEGs) between the control and salt-treated groups were assigned to seven barley chromosomes in two barley subspecies (wild salt-tolerant genotype and ‘Mona’ salt-sensitive cultivar).

Chromosome	Tolerant up-regulated	Tolerant down-regulated	Sensitive up-regulated	Sensitive down-regulated
1H	395	332	86	264
2H	473	499	94	327
3H	485	411	97	255
4H	347	336	69	197
5H	476	504	73	319
6H	304	357	52	219
7H	433	375	84	261
Unassigned	110	178	25	147
Mitochondrion	2	6	0	12
Plastid	0	25	0	29
Total	3025	3023	580	2030

A total of 17,221 differentially expressed isoforms (DEIs) were found in the RNA-Seq analysis. There was a considerable difference between the two barleys for the number of DEIs in a similar trend to the DEGs (see Venn diagrams in Supplementary Figure 2). A greater number of expressed DEIs were produced in the tolerant genotype (12,299) compared with that generated in the sensitive cultivar (4,971 DEIs) under salt stress conditions. Among these DEIs, salt stress upregulated the transcripts of 6,645 and 1,710 DEIs in the tolerant and sensitive barleys, respectively. The frequency distribution of the number of isoforms per gene and their abundance in two barley subspecies (tolerant and sensitive) are given in Supplementary Figure 3. Also, comparing the average number of DEIs per gene showed that about 40% of the expressed genes have a single isoform per gene in the salt-tolerant genotype, while over 60% of the genes have single DEIs in the sensitive cultivar in both control and salt stress conditions. In general, the mean number of DEIs per gene was 2.03 in the tolerant genotype and was 1.9 in the sensitive cultivars. The number of FPKM representing the gene expression abundance was high to very high (from 10 to 100) for the tolerant genotype in salt stress conditions, while it was moderate-to-low (from 1 to 10) in control conditions. On the other hand, the number of FPKM was much less influenced by salt stress in sensitive barley. The noted superiority of wild barley for the DEGs and DEIs compared to barley cultivar was statistically significant at *p* < 0.0001.

#### Gene Ontology and Kyoto Encyclopedia of Genes and Genome Enrichment Analysis of Differentially Expressed Genes

A GO-enrichment analysis was used to categorize the genes enriched in response to salt stress and revealed that the DE genes are significantly enriched (Bonferroni corrected *P* < 0.05) in salt-stress regulated biological processes. Table 3 shows the DEGs significantly enrich 165 GO terms in tolerant genotype and 119 GO terms in sensitive cultivar, respectively. Accordingly, a total of 5,466 DEGs of the wild genotype were assigned to various biological processes, molecular functions, and cellular components (Table 3). A total of 2,244 annotated genes were contributed to the biological functions in the sensitive, which is more than half lower than those in the tolerant barley.

**TABLE 3:** Unique and shared enriched functional categories of DEGs in tolerant (*H. spontaneum*) and sensitive (*H. vulgare*) in response to salt stress annotating by GO term.

Source GO	Term			Tolerant		Sensitive		Effective domain size
	Name	ID GO	Size	*p*-value	Intersection size	*p*-value	Intersection size	
**Shared up-regulated DEGs**								
BP	Response to	0009628	601	0.00000004	109	0.0006	33	19704
	abiotic stimulus							
MF	Protein self-association	0043621	34	0.007	43	0.05	10	22280
**Shared down-regulated DEGs**								
BP	Photosynthesis	0015979	438	0.000000001	173	0.000000001	95	19704
CC	Plastid	0009507	1862	0.000000002	498	0.00000000001	175	19999
MF	Catalytic activity	0003824	11434	0.000000001	1135	0.0000000008	769	22280

**GO**	**Term name**		**ID** **GO**	**Term size**	***p*-value**		**Intersection size**	**Effective domain size**

**Tolerant: uniquely up-regulated DEGs**						
BP	Response to acid chemical		0001101	113	0.000031		30	19704
CC	Spliceosomal complex		0005681	161	0.002		32	19999
MF	Catalytic activity		0003824	11434	0.029		1101	22280
**Tolerant:uniquely down-regulated DEGs**						
BP	Small molecule metabolic process		0044281	1666	0.00003		208	19704
CC	Cytoplasm		0005737	8062	0.000000001		917	19999
MP	Iron–sulfur cluster binding		0051536	198	0.000000002		88	22280
**Sensitive:uniquely up-regulated DEGs**						
BP	Response to abscisic acid		0009737	180	0.008		12	19704
MF	Oxidoreductase activity		0016491	2474	0.0002		66	22280
**Sensitive:uniquely down-regulated DEGs**						
BP	Obsolete oxidation-reduction process		0055114	1956	0.000000002		189	19704
CC	Cell periphery		0071944	2433	0.000000002		233	19999
MF	Protein dimerization activity		0046983	593	0.000000007		88	22280

Gene Ontology enrichment analysis determined gene categories that enriched in response to salt stress (Table 3). Response to the abiotic stimulus (GO:0009628) and protein self-association (GO:0043621) were the top functional groups that were enriched in the shared up-regulated genes between tolerant and sensitive barleys (Table 3). The unique up-regulated genes in tolerant genotype showed significantly enriched catalytic activity (GO:0003824), spliceosomal complex (GO:0005681), and response to chemical (GO:0042221) Whereas, the unique up-regulated genes of sensitive cultivar were significantly enriched for response to abscisic acid (GO:0009737) and oxidoreductase activity (GO:0016491). The shared down-regulated genes between the barleys under salt stress were enriched for photosynthesis (GO:0015979), plastid (GO:0009536), and catalytic activity (GO:0003824). The uniquely down-regulated genes of tolerant genotype showed a small molecule metabolic process (GO:0044281), cytoplasm (GO:0005737), and iron-sulfur cluster binding (GO:0051536) significantly enriched processes. Protein dimerization activity (GO: 0046983), cell periphery (GO:0071944), and obsolete oxidation-reduction process (GO:005511) were the top three processes that were enriched in the unique down-regulated genes of the sensitive cultivar.

**FIGURE 2:**
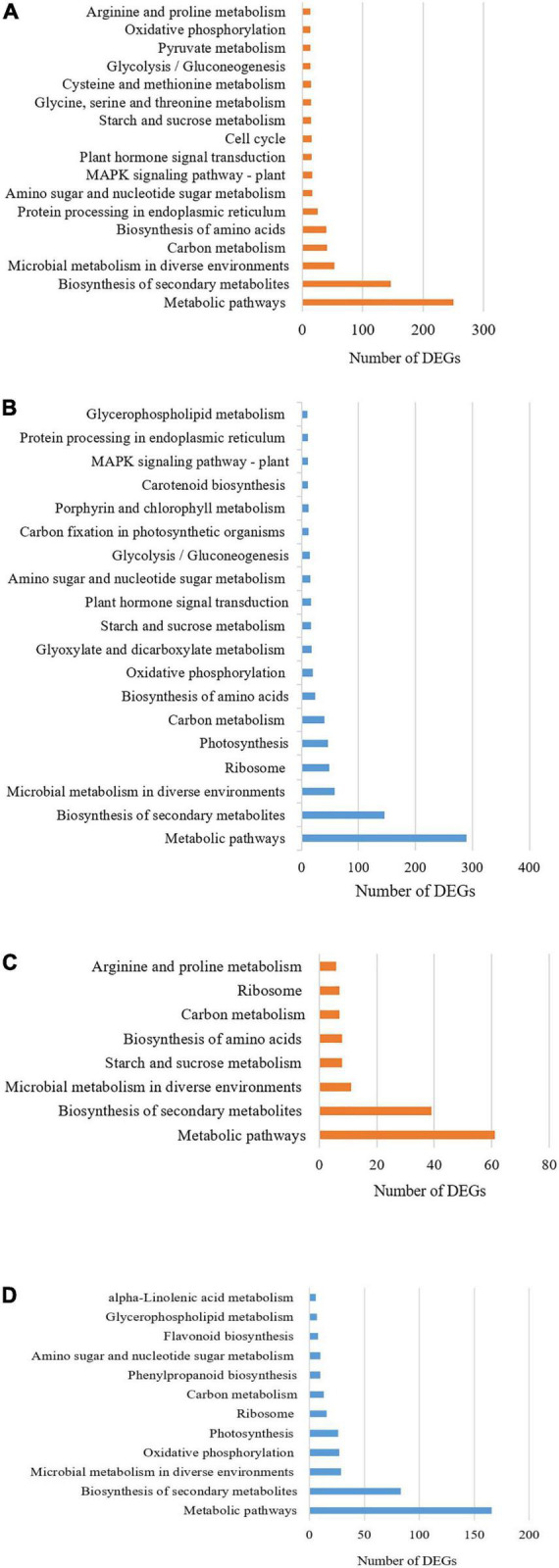
KEGG pathway classification of the differentially expressed genes (DEGs) **(A)** up-regulated and **(B)** down-regulated in salt-tolerant genotype; as well as **(C)** up-regulated and **(D)** down-regulated in sensitive barleys grown under contrasting environments (control vs. salt stress).

Kyoto Encyclopedia of Genes and Genome analysis of the DEGs was carried out to further understand the role that DE genes play in salt tolerance-related pathways. The KEGG pathway enrichment analysis revealed that 902 up-regulated DEGs of salt-tolerant genotype were assigned into 333 pathways and 838 down-regulated DEGs of salt-tolerant genotype were mapped to 327 pathways which seven largest and most important categories enriched in metabolic pathways (KO 01100), biosynthesis of secondary metabolites (KO 01110), carbon metabolism (KO 01200), biosynthesis of amino acids (KO 01230), mitogen-activated protein kinase (MAPK) signaling pathway (KO 04010), plant hormone signal transduction (KO 04075), and phenylpropanoid biosynthesis (KO 00940) (Figures 2A,B).

**FIGURE 3:**
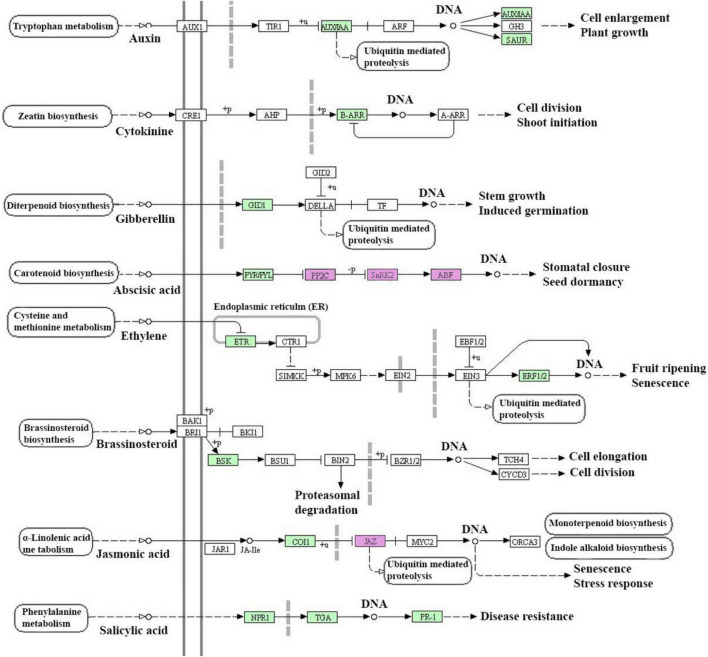
Plant hormone signal transduction detected in response to salt stress conditions (300 mM NaCl) in barley. This figure provides only a KEGG pathway for up-regulated genes in the salt-tolerant genotype and salt-sensitive cultivar. Purple color boxes represent the common differentially expressed genes (DEGs) between salt-tolerant and salt-sensitive barleys and green color boxes represent the specifically DEGs in salt-tolerant genotype. EC number of the encoded enzyme is shown in each box.

**FIGURE 4:**
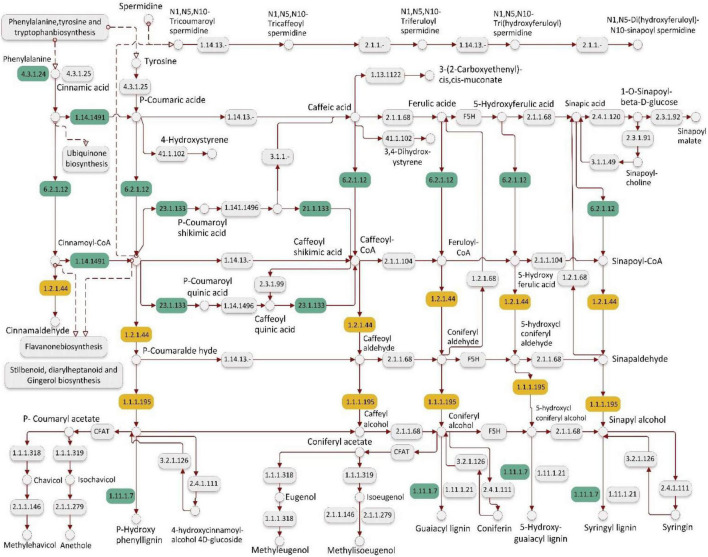
Phenylpropanoid biosynthesis pathway detected in response to salt stress conditions (300 mM NaCl) in barley. This figure represents only one of the KEGG pathway for up-regulated genes in the salt-tolerant and salt-sensitive genotypes. Green color boxes represent the differentially expressed genes (DEGs) in salt-tolerant genotype and yellow color boxes represent the specifically DEGs in salt-sensitive genotype. EC number of the encoded enzyme is shown in each box, e.g., [EC:4.3.1.24]: phenylalanine ammonia-lyase (PAL), [EC:1.14.14.91]: *trans*-cinnamate 4-monooxygenase (CYP73A), [EC:6.2.1.12] 4-coumarate–CoA ligase (4CL), [EC:2.3.1.133]: shikimate *O*-hydroxycinnamoyltransferase (HCT), [EC:1.11.1.7]: peroxidase [EC:1.2.1.44]: cinnamoyl-CoA reductase (CCR), [EC:1.1.1.195]: cinnamyl-alcohol dehydrogenase (CAD).

In the salt-sensitive cultivar, 2610 genes were mapped to the reference common pathway in the KEGG. Among those 169 up-regulated genes were assigned to 164 KEGG pathways and 375 down-regulated genes were assigned to 280 KEGG pathways (Figures 2C,D). The top enriched pathways that up-regulated genes in the sensitive cultivar in response to salt stress included metabolic (KO 1100), biosynthesis of secondary metabolites (KO 01110), starch and sucrose metabolism (KO 00500), and biosynthesis of amino acids (KO 01230). The five top pathways of down-regulated genes in the sensitive cultivar were categorized as: metabolic pathways (KO 1100), biosynthesis of secondary metabolites (KO 01110), oxidative phosphorylation (KO 00190), photosynthesis (KO 00195). Supplementary Figure 4 shows the Venn diagram of the distinct and common KEGG pathways enriched in salt stress conditions. Twenty-three pathways were uniquely and equally up-regulated and downregulated in the tolerant genotype, while lower pathways were uniquely found in the sensitive barley. Plant hormone signal and MAPK signal transduction pathways are shown in Figure 3 and Supplementary Figure 5, respectively. Phenylpropanoid biosynthesis was a common pathway in the two barleys under salt stress conditions (see Figure 4).

### Candidate Genes for Salt Tolerance

The transcripts have been narrowed to focus on the most specifically salt stress-responsive DEGs of wild genotype by comparisons between control and salt stress as well as between the two genotypes. Hence, Table 4 lists the key 80 candidate genes that are associated more specifically with salt tolerance in barley. Supplementary Table 2 presents the same data along with the statistical significance of the expression differences (*p*-value). Among the detected DEGs, the six most important groups include signaling elements, ion transporter, regulatory proteins (transcription factors and kinases), genes involved in ROS scavenging, genes involved in phytohormone biosynthesis, and genes involved in osmotic homeostasis and accumulation of compatible solutes, are described in greater detail below.

**TABLE 4:** Functional annotations of some of the differentially expressed (up- and down-regulated) genes in wild (tolerant) and cultivated (sensitive) genotypes of barley grown under control and salt stress conditions.

Gene ID	Gene function	Gene symbol	Tolerant (Log2 FC)	Sensitive (Log2 FC)
HORVU1Hr1G081310	Snf1-related protein kinase 1	*HvSnRK1 alpha2*	2.007**	0.51^ns^
HORVU4Hr1G056610	Snf1-related protein kinase 1	*HvSnRK1 alpha3*	3.035**	1.08^ns^
HORVU1Hr1G000310	Snf1-related protein kinase 1	*HvSnRK1 beta3*	1.6**	0.53^ns^
HORVU4Hr1G022630	Snf1-related protein kinase 1	*HvSnRK3*	6**	0.32^ns^
HORVU7Hr1G027810	Calcium-binding EF-hand family protein	*HvCML48*	4.6**	0.94^ns^
HORVU3Hr1G109230	Calcium-binding EF-hand family protein	*HvCML31*	6.29**	0.94^ns^
HORVU6Hr1G091790	Calmodulin-binding receptor-like cytoplasmic kinase 1	*HvCaMBP1*	4.31**	0.44^ns^
HORVU6Hr1G072740	Calcium sensing receptor	*HvCaSR*	−3.91**	−0.811*
HORVU2Hr1G101040	Calcium-transporting ATPase	*ATP2* (syn. *PMCA*)	−1.64**	−0.29^ns^
HORVU6Hr1G085890	ATP-binding cassette (ABC) transporter	*HvABCF3*	1.78**	0.52^ns^
HORVU4Hr1G018800	ATP-binding cassette (ABC) transporter	–	0.0^ns^	1.78**
HORVU7Hr1G042800	Potassium transporter	*HvKT24*	4.98**	−0.14^ns^
HORVU7Hr1G109770	Vacuolar proton-ATPase (V-ATPase)	*HvVA68*	2.05**	0.25^ns^
HORVU4Hr1G062880	V-type proton ATPase subunit A	*HvVPH1* (syn. *Hv ATP6V1A*)	2.04**	0.48^ns^
HORVU6Hr1G019930	Protein ABC transporter 1	*HvABC1*	2.05**	−0.22^ns^
HORVU2Hr1G077080	Chloride channel protein CLC-c	*HvCLC-c*	1.50**	1.04*
HORVU3Hr1G068140	Sulfate transporter 3.5	*HvSultr3;5*	8**	1.68^ns^
HORVU2Hr1G100440	High affinity K^+^ transporter	*HvHKT1;2*	2.47**	0.0^ns^
HORVU5Hr1G094840	Cytosolic FE-S cluster assembly factor (family: nitrate, formate, and iron dehydrogenase)	*HvNAR1*	3.58**	0.65^ns^
HORVU6Hr1G029520	Bidirectional sugar transporter	*HvSWEET2A*	2.09**	−0.51^ns^
HORVU3Hr1G082230	Probable magnesium transporter	–	2.81**	−0.37^ns^
HORVU2Hr1G127500	Probable metal ion transporter	–	2.04**	0.71^ns^
HORVU4Hr1G033760	Sodium/hydrogen exchanger 2	*AvNHX1*	1.50**	1.21^ns^
HORVU2Hr1G102840	Vacuolar cation/proton exchanger 2B	*HvCAX2B*	4.02**	−0.73^ns^
HORVU3Hr1G049060	Vacuolar cation/proton exchanger	*HvCAX1A*	1.5**	0.45^ns^
HORVU3Hr1G058300	AKT1 potassium channel	*HvAKT1*	3.72**	1.23^ns^
HORVU7Hr1G040990	SKOR potassium channel	*HvSKOR*	1.71**	2.1**
HORVU6Hr1G070120	Thioredoxin reductase	*HvTrxR1*	2.68**	0.41^ns^
HORVU6Hr1G091330	Thioredoxin-like 3-2, chloroplastic	*HvTrxL3-2*	2.64**	1.07*
HORVU5Hr1G117910	Ferredoxin-3	*HvFdx3*	3.10**	0.59^ns^
HORVU3Hr1G084210	Ferredoxin 3	*HvFdx3*	6.38**	3.19^ns^
HORVU4Hr1G043910	Protein disulfide-isomerase	*HvPDI*	2.26**	1**
HORVU2Hr1G080970	Betaine aldehyde dehydrogenase 1	*HvBADH1*	3.06**	1.10**
HORVU2Hr1G004720	Glycosyltransferase	–	11.38**	2.96^ns^
HORVU2Hr1G019680	Glycosyltransferase	–	8.75**	0.65^ns^
HORVU7Hr1G038510	Glycosyltransferase	–	5.72**	−0.05^ns^
HORVU5Hr1G085310	Glycosyltransferase	–	3.88**	1.04*
HORVU5Hr1G068330	ABA 8′-hydroxylase 2	*HvCYP707A4*	5.15**	−1.28**
HORVU2Hr1G021110	Cu–Zn superoxide dismutase family	*HvSOD1*	1.81**	0.90**
HORVU7Hr1G121700	Catalase	*HvCAT1*	4.22**	0.3^ns^
HORVU3Hr1G074940		*HvPOD*	4.1**	−2.27*
HORVU3Hr1G074960	Peroxidase	*HvPOD*	8.1**	−1.3^ns^
HORVU1Hr1G013950	bZIP domain-containing transcription factor	*HvbZIPx*	2.23**	1.19**
HORVU1Hr1G060810	Gibberellin hormone receptor	*HvGID1c*	2.11**	1.32**
HORVU3Hr1G018860	3-Epi-6-deoxocathasterone 23-monooxygenase	*HvCYP90D1*	2.97**	−0.12
HORVU7Hr1G003170	Lipoxygenase	–	2.4**	0.86^ns^
HORVU4Hr1G005920	Lipoxygenase	*HvLoxB*	2.69**	0.63^ns^
HORVU4Hr1G057210	Ascorbate peroxidase	*HvAPX*	1^ns^	1.55**
HORVU7Hr1G003170	Late embryogenesis abundant protein	*HvLEA18*	6.56**	3.73
HORVU6Hr1G084010	Dehydrin 7	*AvDhn7*	4.24**	7.28
HORVU6Hr1G084070	Dehydrin 4	*HvDhn4*	5.63**	7.37
HORVU6Hr1G012260	HVA22-like protein	*HvHVA22*	1.51**	0.45^ns^
HORVU1Hr1G072780	Delta-1-pyrroline-5-carboxylate synthase	*HvP5CS1*	2.35**	2.16**
HORVU3Hr1G085760	Delta-1-pyrroline-5-carboxylate synthase	*HvP5CSB*	3.44**	4.71**
HORVU1Hr1G080320	Aldehyde dehydrogenase family 12 member A1	*HvALDH12A1*	3.80**	1.43^ns^
HORVU0Hr1G020420	15.7 kDa heat shock protein	*AvHSP15.7*	7.28**	3.88^ns^
HORVU3Hr1G007500	HSP20 family protein	*AvHSP20*	5.38**	2.84
HORVU5Hr1G036590	AP2/ERF-ERF	*HvAp2/ERF*	4.30**	−0.18^ns^
HORVU5Hr1G062940	AP2/ERF-ERF	*HvAp2/ERF*	3.96**	0.28^ns^
HORVU2Hr1G071270	AP2/ERF-ERF	*HvAp2/ERF*	7.43**	−0.42^ns^
HORVU0Hr1G013950	bZIP	*HvbZIP16*	5.83**	3.08^ns^
HORVU5Hr1G106120	bZIP	*HvbZIP63*	2.75**	−0.57^ns^
HORVU2Hr1G119610	MYB	*HvMYB*	3.01**	0.94**
HORVU1Hr1G063740	NAC	*HvNAC56*	4.80**	3.10**
HORVU3Hr1G090920	NAC	*HvNACx*	2.24**	1.74**
HORVU2Hr1G001780	WRKY transcription factor 75	*HvWRKY75*	4.91**	0.59^ns^
HORVU7Hr1G094690	bHLH	*HvbHLH*	4.2^**^	−4.05^**^
HORVU7Hr1G026940	AP2/ERF-ERF	*HvAp2/ERF*	1.78**	1.73**
HORVU6Hr1G008930	RLK-Pelle_LRR-XII-1	–	−5.10**	−0.81^ns^
HORVU5Hr1G119270	RLK-Pelle_WAK	–	8.01**	0.7^ns^
HORVU4Hr1G045160	CAMK2; calcium/calmodulin-dependent protein kinase (CaM kinase) II	–	4.7**	0.06^ns^
HORVU2Hr1G110230	Stress-activated protein kinase 1	*HvSAPK1*	1.20**	2.94**
HORVU4Hr1G057200	Mitogen-activated protein kinase (MAPK)	–	2.2**	0.36^ns^
HORVU4Hr1G006660	MAP3K	–	2.27**	0.24^ns^
HORVU1Hr1G078860	MAP3K	–	4.31**	3.02^ns^
HORVU4Hr1G060940	COI1	*HvCOI1*	1.61**	2.2**
HORVU2Hr1G070880	JAZ	*HVJAZ*	5.73**	−0.014^ns^
HORVU0Hr1G016330	PAL	*HvPAL*	1.9**	−0.058^ns^
HORVU1Hr1G090360	PP2C	*HvPP2C*	4.34**	3.6^ns^
HORVU4Hr1G002330	Na^+^/Ca^2+^ exchanger	*HvNCKX*	2.9**	1.03^ns^
HORVU2Hr1G094160	Beta-carotene 3-hydroxylase	*HvCHYB*	2.9**	−0.32^ns^

*P < 0.05; **P < 0.01; ns, non-significant.

*–, a hitherto un-named gene.*

#### Signaling Factors

Differentially expressed genes encoding calcium-binding protein components (*HvCML31*, *HvCML58*, and *HvCaMBP1*) and protein kinases [*HvSnRK1alpha2*, *HvSnRK1alpha3*, *HvSnRK1beta3*, *HvSnRK3*, and serine/threonine protein kinase (STKs)] were uniquely altered with significant up-regulation in the tolerant genotype. Probable calcium-binding proteins [*HvCML48* (log2 FC = 4.6) and *HvCML31* (log2 FC = 6.29)] were other signaling proteins that their transcripts were highly altered in the tolerant genotype (Table 4). The transcript of calmodulin-binding receptor-like cytoplasmic kinase 1, *HvCaMBP1* (log2 FC = 4.3) was increased, whereas the transcripts of calcium-sensing receptor (*HvCaSR)* and calcium-transporting ATPase (*PMCA*) were reduced in response to salt stress in the salt-tolerant genotype.

**FIGURE 5:**
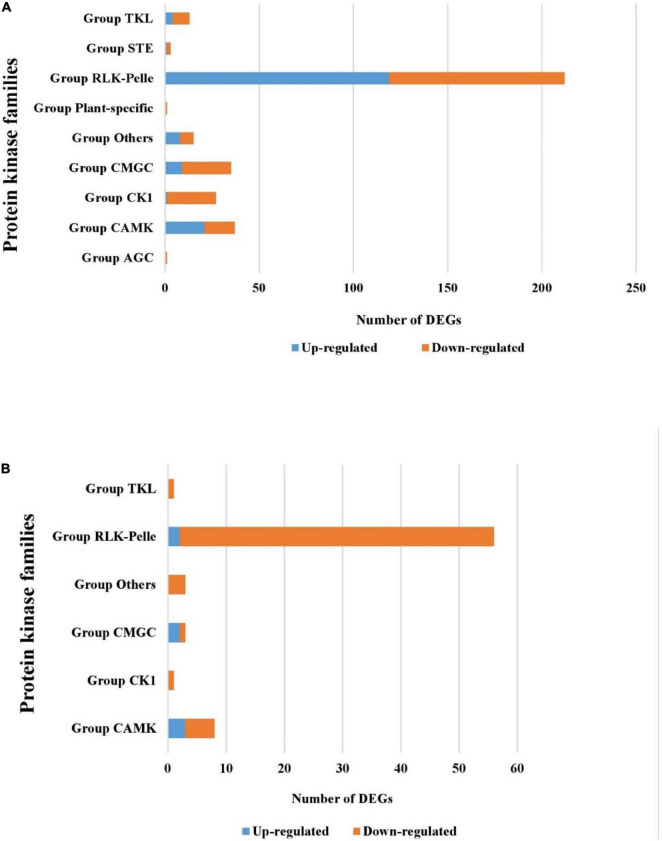
Differentially expressed kinase genes [protein kinases (PKs)] detected in the tolerant wild genotype **(A)** and the sensitive cultivar ‘Mona’ **(B)**.

The analysis of protein kinases (PKs) in the tolerant wild barley showed that 163 up-regulated genes and 155 down-regulated genes encode PKs under salt stress. These PKs are categorized into the following families: AGC, STE, CMGC RLK-Pelle, TKL, and CAMK. The RLK-Pelle family was the largest family containing 119 up-regulated genes found in wild barley (Figure 5A). This family included 27 types of kinases such as RLK-Pelle-DLSV, RLK-Pelle-L-LEC, RLK-Pelle-WAK, and RLK-Pelle-SD-2b. In contrast, only seven and 65 PKs belonging to six families (CAMK, CK1, CMGC, RLK-Pelle, and TKL) were found to be differentially up- and down-expressed under salt stress in ‘Mona’ cultivar, respectively (Figure 5B). Four categories of plant-specific TLK, STE, CKT, and WEE genes were significantly up-regulated only in the wild genotype in response to salt stress. Additionally, one AGC_RSK-2 (Ribosomal S6 Kinases 2) was uniquely down-regulated in the wild barley. The most abundant KP groups were RLK-PELL, followed by CAMK and CMGC which were common in both barleys. In contrast, the transcripts of six MAP3K and one MAPK genes belonging to STE and CMGC families were only up-regulated in wild barley. The expression of TLK, WNK, WEE, NAK, and IRE1 kinase was only altered in wild tolerant genotype as well.

#### Genes Involved in Phytohormone Biosynthesis

*HvCYP707A4* gene that encodes 8-hydroxylase ABA was down-regulated in the sensitive but up-regulated in the tolerant genotype. Another gene in this group, *HvPP2C*, encoding a type C protein phospholipase was differentially expressed in both barley subspecies. In the tolerant genotype, the gibberellin hormone receptor, *HvGID1c*, was significantly induced under salt stress, while the differential expression was not significant in the sensitive cultivar. Salt stress was only induced significantly in the *HvCYP90D1* gene, in the tolerant genotype encoding 3-Epi-6-deoxocathasterone 23-monooxygenase that involves brassinosteroid biosynthesis. Two genes encoding lipoxygenase (*HvLoxB* and unknown) were shown to be up-regulated significantly in the salt-tolerant but not in the sensitive genotype. In the tolerant barley, *HvJAZ* and *HvCOI1* genes were up-regulated in the jasmonic acid signal transduction pathway.

#### Transcription Factors

**FIGURE 6:**
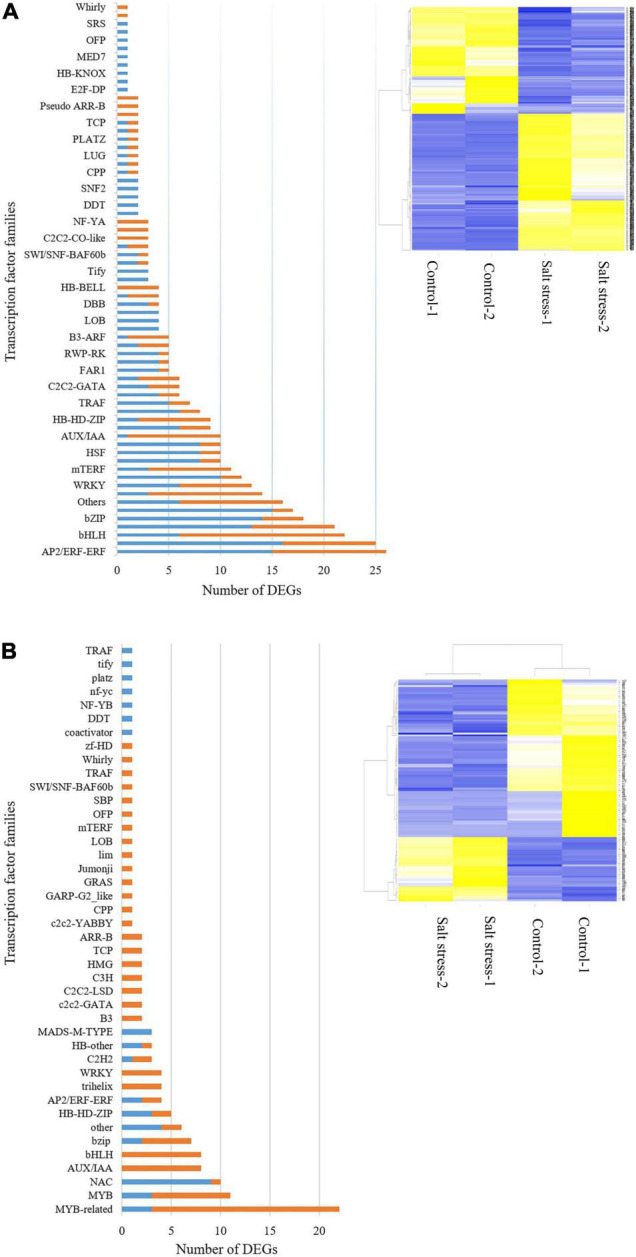
Transcription factors (TFs) differential expressed. Number of up (blue bar)- or down (orange bar)-regulated gene in transcription factors (TFs) expressed (left) and heat map of expression changes in differentially expressed genes (DEGs) under control and salt -stress conditions (right) in the **(A)** salt-tolerant genotype and **(B)** salt-sensitive cultivar (‘Mona’).

In the salt-tolerant genotype, 69 transcription factor (TF) families containing 399 genes were identified, 177 of which were down-regulated and 222 up-regulated in response to salt stress using the iTAk pipeline. In this study, MYB, AP2/ERF-ERF, MYB-related, NAC, bZIP, WRKY, bHLH, Trihelix families were profoundly expressed in salt stress compared with control conditions. Those TF families that were downregulated in response to salt stress included bHLH, GNAT, AUX/IAA (Figure 6A).

In contrast to the tolerant genotype, the ‘Mona’ cultivar showed much lower TFs differentially expressed (83: 24 up- and 59 down-regulated, belonging to 37 TF families). NAC, HSF, MADS-M-TYPE, HB-HD-ZIP, MYB families were mainly up-regulated, while MYB, bHLH, AUX/IAA, trihelix, and WRKY followed a down-regulation pattern in this cultivar (Figure 6B). *HvbZIPx. HvbZIP63*, and *HvbZIP16* belonging to the bZIP family were up-regulated in the tolerant barley, but not significantly impacted in the sensitive barley. *HvMYB* and *HvWRKY75* genes were also induced under salt stress in the tolerant genotype, while no expression changes were observed in the sensitive cultivar. In salt-tolerant genotype, *HvAp2/ERF* genes with log2 FC > 3 were identified but not detected in the ‘Mona’ cultivar. *HvNAC56*, *HvNACx*, and *HvbHLH* were the common TFs induced by salt stress in the two barleys.

#### Ion Transporters and Channels

Comparative transcriptome analysis of transporters and channels of wild (tolerant) and cultivated (sensitive) barleys in control and salt stress conditions revealed several crucial genes involved in the ion transport and channels. Tolerant genotype showed an upregulation of the transcripts of the following genes: *HvABCF3*, *HvABC1, HvVA68, HvVPH1*, *HvCLC-c*, *AvNAR1, HvSultr3;5, AvNHX1, HvCAX1A, HvCAX2B, HvKT24*, *HvHKT1;2, HvSKOR*, five V-type proton- transporting ATPase (V-ATPase) genes, and probable magnesium transporter (a hitherto un-named gene). The expression of *HvABC1 and HvABCF3* was almost 1.8 folds higher in two barleys when they were grown under 300 mM NaCl as compared to control conditions. *HvVA68, HvVPH1*, and five V-ATPase genes were uniquely expressed in the tolerant barley in response to salt stress. The chloride channel gene, *HvCLC-c*, was upregulated following NaCl treatment in both barleys. Wild tolerant barley showed 8-fold increases in the *HvSultr3;5* transcripts due to salt stress while not inducing significantly in the ‘Mona’ cultivar.

The expression of the genes belonging to the transporter families of low-affinity K^+^ transporter (AKT), high-affinity K^+^ transporter (HKT), HAK/KUP/KT potassium, and Na^+^/H^+^ exchanger (NHX) was significantly up-regulated by salt stress specifically in the tolerant barley. These can be listed as follows: *HvNHX1*, *HvAKT1*, *HvHKT1;2*, and *HvKT24.* The expression of *HvCAX1A, HvCAX2B, HvNCKX*, and two probable magnesium transporter genes (hitherto un-named) was also significantly induced by salt stress specifically in the tolerant barley. In contrast, in response to salt stress, the expression of the potassium channel gene, *HvSKOR*, was differentially up-regulated in two barleys. *AvNAR1* was expressed differentially in tolerant genotype while its differential expression in the sensitive cultivar was not significant. These overall results reveal that most of the genes encoding the ion transporter and channel proteins were specifically expressed in the tolerant barley in salt stress conditions.

#### Genes Encoding Reactive Oxygen Species Scavenging Proteins

The expression of *HvTrxR1* and *HvTrxL3-2* (thioredoxin reductase and chloroplast thioredoxin respectively or NTRs) genes, as well as duplicate ferredoxin (*HvFdx3*) genes and glycosyltransferases, was significantly induced in the tolerant barley under stress. In addition, the genes encoding enzymatic antioxidants in plants include catalase (CAT), ascorbate peroxidase (APX), and superoxide dismutase (SOD) symbolized *HvCat1*, *HvSOD1*, and *HvAPX*, respectively, were significantly upregulated in the salt-tolerant barley, but only *HvAPX* gene was upregulated significantly in the ‘Mona’ cultivar. *HvPDI* gene encoding the protein disulfide isomerase, a multifunctional enzyme that mediates the isomerization through disulfide bonds, catalyzes the cysteine-based redox reactions, and assists in the acquisition of the correct three-dimensional structure of the protein was up-regulated in two barleys. *AvBADH1* gene encoding a form of the beta-aldehyde dehydrogenase (BADH) enzyme up-regulated approximately three-fold in the tolerant and almost one-fold in the sensitive barley, both of which were statistically significant (*P* < 0.01). Phenylalanine ammonia-lyase (*HvPAL*) gene involved in salicylic acid synthesis expressed under salt stress in the tolerant genotype while it was down-regulated in response to salt stress in the sensitive cultivar.

#### Regulation of Genes Involved in Osmotic Homeostasis and Accumulation of Compatible Solutes

Both barleys had significantly elevated expression of the *HvLEA18*, *AvDhn7*, *HvDhn4*, *HvBADH1*, *HvP5CS1*, and *HVP5CSB* genes, while the *HvHVA22* gene was significantly upregulated specifically in the tolerant genotype. Heat shock proteins (HSPs) are other cell-protective molecules that may be functionally relevant to this category. Several HSP transcripts showed expression changes, the two most significant and differentially expressed in both barleys were *AvHSP15.7* and *AvHSP20* genes. Indeed, in addition to the role played as a source of carbon and energy, sugars, also function as osmotic regulators to alleviate abiotic stresses in plants that the *HvSWEET2A* gene was upregulated in salt-tolerant genotype.

### Proposed Salt Tolerance Mechanisms

**FIGURE 7:**
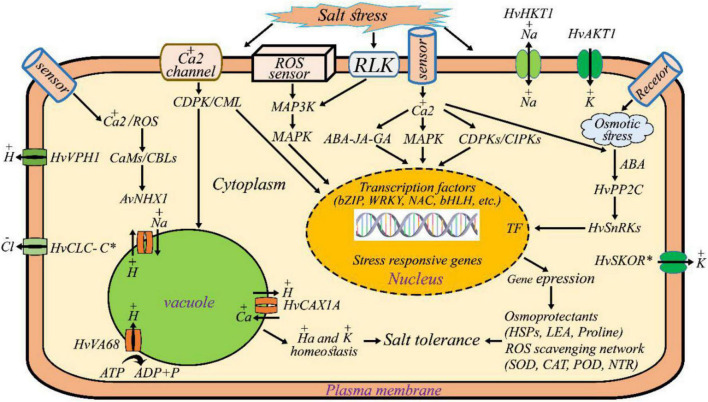
Salt tolerance mechanisms of wild barley (*H. vulgare* ssp. *spontaneum*). Wild barley maintains lower cellular Na^+^ content by activating ion transporters such as NHXs (sodium exchanger), CLC-C (chloride channel protein), SKOR (outward rectifying K^+^), AKT (inward rectifying K^+^), HKT (high affinity potassium transporters) and CAX (vacuolar cation/proton exchanger). Thus, ion transporters associate with maintaining K^+^/Na^+^ ratio in salt tolerant (wild) plant. Vacuolar H^+^-ATPase establishes a proton gradient across the vacuolar membrane that drives vacuolar Na^+^/H^+^ exchanger activity. Several key components including kinase (e.g., MAPK, MAP3K, CDPKs) activated in signal perception, transduction and amplification. Transcription factors (e.g., NAC, bZIP, bHLH, WRKY), detoxification and ROS scavenging enzymes [catalase (CAT), superoxide dismutase (SOD), and ascorbate peroxidase (APX), peroxidase (POD), thioredoxin reductase (NTR)], hormones (ABAs), osmoprotectant [late embryogenesis abundant (LEA), heat shock proteins (HSPs), and proline] and some other salt-tolerance related mechanisms were specifically induced to alleviate osmotic stress, to mediate ion homeostasis and/or to modulate oxidative stress.

Figure 7 summarizes our findings of the underlying adaptive mechanisms by which the wild barley cells sense, and deploy interconnected molecular pathways in response to salt stress. We look at these pathways briefly below. Wild barley activates ion transporters such as NHXs, CLC-C, SKOR, AKT, HKT, and CAX for maintaining the homeostasis of Na^+^ and K^+^ ions in the cytosol. The Na^+^, K^+^ -ATPase maintains Na^+^ and K^+^ gradients across cell membranes by pumping Na^+^ ions out of cells while bringing K^+^ ions in. Vacuolar H^+^-ATPase establishes a proton gradient across the vacuolar membrane that drives vacuolar Na^+^/H^+^ exchanger activity. Several key components including kinase (e.g., MAPK, MAP3K, CDPKs) activated in signal perception, transduction, and amplification. Transcription factors (e.g., NAC, bZIP, bHLH, WRKY), detoxification and ROS scavenging enzymes [catalase (CAT), superoxide dismutase (SOD), and ascorbate peroxidase (APX), peroxidase (POD), thioredoxin reductase (NTR)], hormones (ABAs), osmoprotectant (late embryogenesis abundant (LEA), heat shock proteins (HSPs), and proline and some other salt-tolerance related mechanisms were specifically induced to alleviate osmotic stress, to mediate ion homeostasis, and/or to modulate oxidative stress.

### Real-Time Expression Analysis of Salt Responsive Genes

**FIGURE 8:**
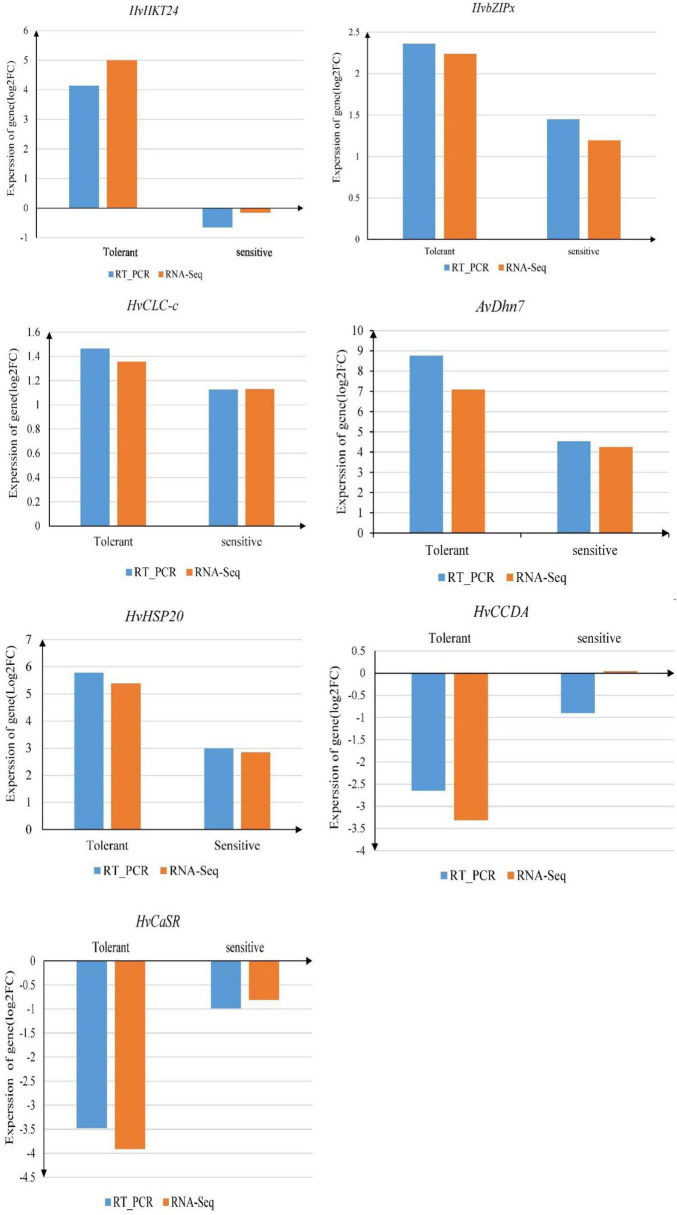
Relative expression of seven genes (*HvKT24*, *HvbZIPx*, *HvCLC-c*, *AvDhn7*, *HvHSP20*, *HvCCD*, and *HvCaSR*), which were also detected by RNA-Seq, using qRT-PCR.

The expression profiles of seven genes randomly selected from the significant DEGs were used for real-time PCR analysis from which a comparison was made with their corresponding transcriptome data. Six out of the mRNA transcripts generated in response to salt stress by qRT-PCR appeared to follow a similar pattern as in the RNA-Seq (Figure 8). Five of the stress-responsive genes namely *HvHKT1;2*, *HvKT24*, *AvDhn7*, *AvHSP20*, *HvbZIPx*, *HvCCDA* were up-regulated while the two remaining genes, *HvCLC-c* and *HvCaSR*, were down-regulated in response to salt stress. There was only one inconsistency between qRT-PCR and RNA-Seq data with an inverse regulation pattern for the *HvCLC-c* gene that was down-regulated in qRT-PCR and up-regulated in transcriptome results.

## Discussion

### Plant Growth and Physiological Attributes

Malondialdehyde (MDA) is a product of polyunsaturated fatty acid peroxidation. Salt stress caused an increase in the leaf MDA content of the tolerant genotype, while this increase was much higher for the salt-sensitive barley. This result indicates a higher rate of lipid peroxidation in the sensitive than tolerant genotype under salt stress conditions. These are in good agreement with previous results obtained by Kiani et al. (2015) on *Aegilops cylindrica* with a greater MDA content in the sensitive than the tolerant genotype. The destructive effect of salt stress on lipid peroxidation has already been shown by Zeeshan et al. (2020) who found a higher MDA content in salt-tolerant than salt-sensitive genotype and that MDA can potentially be used as an indicator of salt tolerance in wheat and barley. Nefissi Ouertani et al. (2021) also observed an association between low MDA content and higher salt-stress tolerance in barley.

The RWC was decreased in response to salt stress in both sensitive and tolerant genotypes, but the decrease in RWA was more pronounced in the sensitive cultivar. Reduction of relative water content due to salt stress has already been observed in barley (Ebrahim et al., 2020), which is in line with the results of this study. Comparison of MSI results between tolerant and -sensitive genotypes shows the higher membrane damage and electrolyte leakage in the sensitive than the tolerant barley. This finding is consistent with that of Ebrahim et al. (2020) for salt stress and Bahrami et al. (2019) for heat stress in barley. Salt stress causes damage to the plasma membrane and cell leakage and a rapid decrease in cytosolic nutrients, so maintaining membrane integrity is essential to prevent membrane leakage. Wild barley was also defined as being superior to cultivated barley for salt tolerance using STI. A recent report shows that STI is the most effective discrimination index for screening salt-tolerant genotypes, and STI for grain weight is governed by several QTL with mainly additive effects in barley (Sayed et al., 2021).

Lower Na and K concentrations along with a higher K/Na ratio found in the tolerant genotype compared with the sensitive genotype, indicate the different mechanisms by which wild and cultivated barleys may have evolved their own specific interactions with salt stress. Because these two sub-species are distinct from each other, one may speculate that a combined Na^+^ extrusion from the root cells with enhanced salt exclusion from the shoots might be interpreted in favor of wild barley. The distinctive salt tolerance of wild barley can be seen in the context of the predictability of evolutionary responses in wild and cultivated grasses, as a potential support to the already hypothesized evolutionary strategy in saline-adapted wild plants. Consistent with this line of reasoning, Ebrahim et al. (2020) demonstrate that plant survivability in salt-tolerant wild barley was negatively associated with Na concentration in the root and leaf and positively related with the leaf K/Na ratio. Carden et al. (2003) compared salt tolerance of two cultivated barleys at 5 and 8 days of NaCl stress and found that tolerant cultivar has much lower root cytosolic Na^+^ content than sensitive cultivar at 5 days, with an indifferent effect at 8 days.

### Transcriptome Analysis

One of the important implications of this study is the clear link between the overall RNA-Seq dataset and those of the qRT-PCR analysis, which enabled us to establish a relationship between the DEGs and salt-stress tolerance as well as to identify key genes associated with salt tolerance in barley. Though sampling artifacts can be ruled out as the RNA samples used for both experiments were the same, other explanations could be put forward to justify the observed inconsistency between up-regulation of *HvCLC-c* in the transcriptome experiment and its down-regulation in the qRT-PCR. First, CLC chloride channels have a large gene family in eukaryotes whose members often exhibit distinct expression patterns (Teakle and Tyermam, 2010). Second, consequently, there exist an enormous number of variants of the alleles and the challenge is to navigate through and identify any that are likely to match the primer sequence designed. Therefore, it can be assumed that the RNA-Seq data is more reliable since the qRT-PCR vastly depends on the primer sequence.

During plant salt stress responses, the tolerance mechanisms are regulated by complex cross-talking signaling and transduction pathways (Mahajan et al., 2008; Hasanuzzaman et al., 2018). Indeed, the pictures emerging from our DEGs data suggest that MAPK signaling pathways, calcium signaling pathways, and hormone signal transduction pathways regulate the expression of a network of TFs mediating salt-stress responses in barley. These findings are similar to Goyal et al. (2016), who in a transcriptomic study in wheat found the hormone signal transduction was associated with salt tolerance). The number of DEGs and DEIs found in salt stress conditions was significantly higher than those in normal conditions in two barleys. These findings are in agreement with earlier transcriptome studies in barley both in salt stress (Zhu et al., 2020) and in drought stress conditions (Harb et al., 2020). The number of DEGs has observed in salt-tolerant barley in our study was about five-fold higher than those found for a salt-tolerant line by Zhu et al. (2020). This is consistent with our results and implies that wild subspecies of barley as a tolerant genotype had a very much stronger response to salt stress than the domesticated genotype.

Our transcriptome results are fully consistent with our empirical physiological data and therefore point to the stronger expression of the responsive genes in the tolerant than the sensitive barley. It is interesting to note that the DEGs yielded approximately three-fold greater DEIs in the salt tolerant barley. This finding supports a well-established understanding that alternative pre-mRNA splicing can be induced by environmental stresses and plays a crucial role in shaping the transcriptome of plant cells (Laloum et al., 2018). As such, transcriptional regulation is highly plastic, depending on the presence of transcription factor proteins (TFs) and their binding to short DNA binding motifs (Matys et al., 2003). Accordingly, some of the important elements and functional implications of the salt tolerance strategies of barley are discussed below.

#### Signaling Elements

Plant cell membranes are crucial players in cell functioning such as structural integrity and transport, as well as sensing and response to environmental and biological stimuli. Cellular signal transduction networks are composed of various proteins such as sensor, transducer, and effector proteins, which include receptors, adaptor proteins, kinases, TFs, and secondary messengers like Ca^2+^, H_2_O_2_, NO, and ABA. In the two examined barleys, the *HvCaSR* transcript was weakly (small) or strongly (large) down-regulated on day 6 after 300 mM NaCl treatment in sensitive and tolerant genotypes, respectively. A plasma membrane GPCR (G protein-coupled receptor) also named CaSR (calcium-sensing receptor) is involved in regulating intracellular Ca^2+^ ion homeostasis and as a probable messenger outside cells (Hofer and Brown, 2003). The CaSR receptor is induced not only by Ca^2+^, but also by other divalent and trivalent cations, polyamines, amino acids, and other polycationic ligands. It is coupled to many different intracellular signaling cascades through heterotrimeric G proteins. The CaSR also plays a pivotal role in the modulation of various cellular processes, such as apoptosis, proliferation, differentiation, chemotaxis, secretion, and ion channel activity (Hofer and Brown, 2003). Having the above definitions in mind, we can postulate two reasons for the down-regulation of *HvCaSR* in our study. First, the reproducibility of the expression of *HvCaSR* has been confirmed by two independent experiments. Second, salt stress causes an influx of Ca^2+^ across the plasma membrane, which results in a calcium-depleted extracellular environment.

In the current study, calcium sensors including calmodulins (CaMs), calcineurin B-like proteins (CBLs), calcium-dependent protein kinases (CDPKs), and calmodulin-like proteins (CMLs) have been widely documented (Hofer and Brown, 2003; Mahajan et al., 2008). The current study findings, therefore, give further evidence for the functional role of the *HvCML31*, *HvCML58*, and *HvCaMBP1* genes in triggering wild barley tolerance to salt stress. Then, several key components including kinase (receptor-like kinase/Pelle, RLK-Pell) activated in signal perception, and the other kinases are involved in signal transduction and signal amplification (Lehti-Shiu and Shiu, 2012). The results are consistent with previous reports in that a large number of RLK-Pelle proteins were associated with biotic and abiotic stresses in Arabidopsis, rice, and barley (Harb et al., 2020). In addition, in the tolerant genotype, CDPKs (Ca^2+^ dependent protein kinases) and SNF1-related kinases (SnRKs) are among the salt-induced specific genes belonging to the CAMK group that activated in ABA-independent and ABA-dependent signaling pathways through phosphorylation of ion channels and transducers and simultaneously regulate stress response gene expression (Kitsios and Doonan, 2011; Lehti-Shiu and Shiu, 2012). Exposure of salt-tolerant barley cells to salt stress leads to activation of MAPK cascades composed of MAPKs and two MAPK kinases (MAP3Ks) indicating their important role in salt-stress signal transduction. The GO annotation and KEGG pathway analysis suggested that most of the barley DEGs were involved in salt signaling and transduction pathways fall into two functional categories: MAPK signaling pathways and hormone signal transduction pathways. Phytohormones have been discussed as distinct entities in a separate section below.

#### Phytohormone Biosynthesis

Phytohormones are crucial signaling molecules that regulate growth, development, and defense in plants. The multiple hormone biosynthesis-related DEGs found in this study show their function as regulatory factors of adaptation and survival in the face of diverse environmental stresses. In rice, Formentin et al. (2018) found a large number of the expressed genes that were associated with hormone regulation in response to salt stress. Key enzymes in ABA catabolism, ABA 8’-hydroxylases, are encoded by *CYP707A*. The special expression of the *CYP707A* gene in the tolerant genotype may suggest that this gene plays a central role in regulating tolerance response through various mechanisms in wild barley. This finding is consistent with the reported data indicating the enhanced expression levels of *CYP707A* in soybean under salt and drought stress conditions (Zheng et al., 2012). Protein phosphatase 2C (PP2C) is another ABA-related gene that is known to participate in the stress responses in eukaryotes including plants (Sheen, 1998). These results are in accordance with those reported in wild maize (*Zea mays* ssp. *mexicana* L.) and show the up-regulation of four candidate *PP2C* genes in response to cold and drought stress (Lu et al., 2017). Given that *HvPP2C* is inducibly expressed and together with *SnRK2s* and *HvbZIPx* gene*s* are critical components of hormone signal transduction and MAPK signaling pathway. They would likely inhibit water loss and osmotic stress through stomata closure. Hence, the *PP2C* gene family is a player in ABA signaling transduction that assist the plant in tolerating both salt and water stress. The expression of the gibberellic acid receptor gene, *HvGID1c*, was up-regulated in both barleys due to salt stress. This is in line with a recent report that also found higher expression of the *GID1* gene in response to salt stress in millet (Yuan et al., 2021).

CYP90D1 (encodes a cytochrome P450 gene) and thioredoxins (aforementioned) involved in brassinosteroid biosynthesis were up-regulated in the tolerant genotype due to the product and presence of the brassinosteroid hormone. Brassinosteroids (BRs) are steroid hormones that act as signal molecules to activate defense response under stress conditions (Ahammed et al., 2020). CYP90D1 was significantly up-regulated in Arabidopsis in response to drought stress (Li et al., 2020). Two different genes encoding for lipoxygenase up-regulated in both barleys but more specifically in the salt-tolerant barley. These are associated with the oxygenation of fatty acid and the formation of fatty acid hydroperoxide, which ultimately lead to the jasmonic acid synthesis in the octadecanoic pathway. Consistent with our findings, Zhang et al. (2017) found that lipoxygenases genes involved in jasmonic acid signaling were up-regulated by salt stress in sweet potato. Likewise, eight genes encoding lipoxygenase were up-regulated significantly in finger millet under salt stress (Rahman et al., 2014). In the tolerant barley, *HvJAZ* and *HvCOI* genes were up-regulated due to salt stress. These genes are associated with the jasmonic acid signal transduction pathway and their expression alterations were reported in *Arabidopsis thaliana* (Ali and Baek, 2020).

#### Transcription Factors

After perceiving the stress signal, transcription factors (TFs) are activated. We detected significant up-regulation of AP2/ERF-ERF, bZIP, MYB-related, WRKY, Trihelix, and bHLH TFs only in the tolerant genotype which suggests the crucial roles of these TFs in regulating transcription of the downstream genes responsible for tolerance to salt stress. On the other hand, other TFs like NAC, HSF, MADS-M-TYPE, HB-HD-ZIP, and MYB were differentially expressed in both barleys. These findings in turn suggest the involvement of these TF groups in the type of adaptive responses that underlying tolerance mechanisms are shared between the two barleys. Some of these TF results are consistent with an earlier report examining the transcriptome response of a salt-tolerant cultivar to salt stress in wheat (Amirbakhtiar et al., 2019). Our results also agree well with those found in other crop species such as oat (Wu et al., 2018), finger millet (Rahman et al., 2014), sorghum (Cui et al., 2018), maize (Mittal et al., 2018), and alfalfa (Lei et al., 2018). In the current study, salt-induced genes encoding AP2/ERF-ERF, bZIP, MYB-related, WRKY, Trihelix, and bHLH proteins were only upregulated in the tolerance genotype. In wild diploid species of cotton, salt stress specifically affected AP2, bZIP, bHLH, MYB, NAC, and WRKY families at 12 to 24 h after stress, while most of the known TFs had significantly expressed at 96 to 142 h after stress (Zhang et al., 2016).

#### Ion Transporters and Channels

The precisely regulated influx and efflux of Cl^–^ and Na^+^ ions across the cell and organelle membranes are essential for plant salt tolerance (Arzani and Ashraf, 2016). This action is performed by three membrane-embedded proteinous structures known as ion channels, ion pumps, and ion transporters (Teakle and Tyermam, 2010; Hedrich, 2012; Nedelyaeva et al., 2020). What do our transcriptome data suggest about how salt-tolerant wild barley performs the role of regulating the expression of transcripts capable to act in simultaneous multiple ion transporters and channels?

The “chloride channel” (CLC) family is comprised of both active transporters and passive channels. In *Arabidopsis thaliana*, the CLCc transporter was found to be located in vacuolar membranes (Nedelyaeva et al., 2020). *HvCLC-c* gene up-regulated under salt treatment in both wild and cultivated barleys but with a stronger expression in wild barley. The observed suggest that increased expression of CLC ion channels genes under stress is necessary for chlorine homeostasis and modulates salt tolerance response.

Indeed, a growing number of studies demonstrate a crucial role for Na^+^ transporters in cultivated and wild plants, which decrease the toxic levels of Na^+^ ions and stabilize the cytosolic ion homeostasis during salt stress. Na^+^ -mediated ion transporters (i.e., ion cotransporters, ion antiporters, and ion exchangers) including high-affinity potassium transporters (HKTs), KUP/HAK/KT K^+^ (HAK) transporter, low-affinity K^+^ transporter (AKT), stelar K^+^ outward rectifier (SKOR) and Na^+^/H^+^ exchanger (NHX) as well as stelar K^+^ outward rectifier (SKOR) channel were the major Na^+^ transporter family genes that observed in our transcriptome data. In barley, Han et al. (2018) demonstrated that *HvHKT1;1* not only is a critical transporter of Na^+^ but also associated with Na^+^ retrieval from xylem, both of which result in a decrease in Na^+^ accumulation in shoots. In barley, Amarasinghe et al. (2019) reported that the *HvHKT1;5* and *HvHKT2;1* genes were only expressed in the root tissues, while, the expression of *HvHKT1;2* was induced in all plant organs, but predominantly in the leaf blade and sheath tissues. We did not trace the root transcripts, so it may not be surprising that *HvHKT1;5* and *HvHKT2;1* have not been identified in our study.

In response to salt stress in wild barley, potassium channel and transporters genes including *HvKT24*, *HvHKT1;2, HvAKT1*, and *HvSKOR* were induced. Similar observations have been made in rice that some K^+^ transporters (HAK5 and HKT) were upregulated (Walia et al., 2007). In millet, three KUP genes of potassium transporters were up-regulated in salt treatment (Rahman et al., 2014). Sulfur atoms in cysteine residues are highly sensitive to oxidation and such oxidative alterations often play a key role in the localization, structure, and function of the protein. Sulfur and its derivatives play role in scavenging the free radicals under different abiotic stresses (Hasanuzzaman et al., 2018). In our study, a Mg^2+^ transport gene and two Ca^2+^/H^+^ exchanger genes (*HvCAX2B* and *HvCAX1A*) were up-regulated under salt stress in the wild tolerant barley which indicates the essential function of Mg^2+^ and Ca^2+^ transporters in salt tolerance of barley. Increased expression of the *CAX* gene has been observed in sweet potato (Zhang et al., 2017). Similarly, the role of *CAX* in the improvement of salt tolerance has been established using transgenic plants in Arabidopsis (Luo et al., 2017). Genes belonging to the cation/proton exchanger group were present in both plasma membranes and vacuole membranes, and are an important group in creating ionic homeostasis (Luo et al., 2017). The K^+^-dependent Na^+^/Ca^2+^ exchanger (*HvNCKX*) gene was upregulated under salt stress in the tolerant barley. This gene plays an important role in calcium homeostasis (Emery et al., 2012). The over-expression of this exchanger might be as a result of homeostatic compensation in Ca^2+^ signaling, brought about by other Ca^2+^ transporters such as *HvCAX1A*, *HvCAX2B* in the tolerant barley.

In the salt-tolerant barley, vacuolar ATPases (V-ATPases) and plasma membrane ATPases were differentially upregulated. The active Na^+^ ion efflux generally occurs through ion channels and ion transporters located in the tonoplast and plasma membrane. Therefore, enhanced activity of the tonoplast and plasma membrane ATPases are associated with salt-adaptation as the energy source for pumping out of Na^+^ ions across the cell wall and sequestration of Na^+^ ions in the vacuole (Mansour et al., 2003; Zhang et al., 2011; Arzani and Ashraf, 2016). In addition, the expression of the *HvNHX1* transporter was increased in the leaves of salt-stressed tolerant barley. The Na^+^/H^+^ exchangers (NHXs) lessen the Na^+^ ions accumulation in the cytosol by compartmentalization of excess Na^+^ ions in the vacuole (Arzani and Ashraf, 2016). In line with the current study, the important role of *HvNHX1* in salt tolerance has been emphasized by Saade et al. (2018) who found a significantly higher increase in transcript level of *HvNHX1* of plants exposed to 200 mM NaCl than control plants in barley. ABC transports have been reported to be involved K/Na homeostasis in Arabidopsis and improve salt tolerance (Mansuri et al., 2019). Sulfur also interacts with the phytohormones such as ABA, auxins, cytokinins, gibberellins, jasmonic acid, and salicylic acid ethylene under abiotic stress conditions, thereby potentially regulating plant defense. The sulfate flux (SO4^2–^) into the cell controls by the sulfate transporters. The expression enhancement of sulfate transporter gene *HvSultr3;5* in wild barley is likely associated with salt stress tolerance. In plants, *Sultr3;1* gene expression under salt stress has already been noted (Gallardo et al., 2014). Kataoka et al. (2004) described the *Sultr3;5* as a key sulfate transport component that accelerates the root-to-shoot transport of sulfate which is localized in the root’s pericycle cells and xylem parenchyma in *Arabidopsis thaliana*.

#### Genes Encoding Reactive Oxygen Species Scavenging Proteins

An imbalance between oxidants and antioxidants in favor of the oxidants, known as oxidative stress, results in a disruption of redox signaling and control and/or damage to molecular components such as DNA, protein, and lipid with ultimate cell dysfunction or death. ROS also triggers a signaling cascade such as the MAPK to activate redox-sensitive TFs (Fichman et al., 2019; Huang et al., 2019). Salt stress leads to a significant ROS accumulation with destructive oxidative damages. The results of the current study show that the production of free radicals was increased during the salt stress, but was effectively quenched by a robust antioxidant defense system in the salt-tolerant barley. The expression of genes encoding enzymatic antioxidants including *HvSOD1*, *HvAPX*, *HvCat1*, *HvPOD*, thioredoxin reductase (*HvTrxR1*, *HvTrxL3-2* genes or NTRs), *HvPDI*, and ferredoxin (*HvFdx3*) has specifically occurred in the tolerant barley under salt stress. These are consistent with the findings that genes encoding peroxidases and catalase were induced in response to salt stress in finger millet (Rahman et al., 2014). It is well known that non-enzymatic and enzymatic antioxidants play a pivotal role in scavenging ROS and improving tolerance to abiotic stresses in the plant kingdom (Huang et al., 2019).

In the tolerant genotype, expression of the genes encoding PAL: phenylalanine ammonia-lyase, BCH: beta-carotene 3-hydroxylase, HPT: homogentisate phytyltransferase/homogentisate geranylgeranyl transferase was increased following salt stress. These genes are respectively involved phenylpropanoid biosynthesis, ubiquinone and other terpenoid-quinone biosynthesis, and carotenoid biosynthesis pathways. These enzymes catalyze various reactions that yield products of non-enzymatic antioxidants such as α-tocopherol, zeaxanthin, zeinoxanthin, and flavonoid. The expression of *HvPAL* gene, which encodes phenylalanine ammonia-lyase enzyme, was enhanced in salt-tolerant barley, while it was down-regulated in the sensitive one due to salt stress. This result is in line with a previous study on finger millet that shows an increase in the expression of this enzyme in the tolerance genotype and a decreased expression in the sensitive one (Rahman et al., 2014). PAL catalyzes the first reaction in the pathway of general phenylpropanoid resulting in the production of the phenolic compounds in plants. Phenolic compounds are non-enzymatic antioxidants involved in the scavenging of ROS and have potential implications for plant stress tolerance mechanisms (Kiani et al., 2021). The beta-carotene 3-hydroxylase gene (*HvCHYB*) involved in carotenoid biosynthesis showed increased expression. Expression of this gene in Arabidopsis increases the conversion of beta-carotene to xanthophyll and in turn enhances tolerance to osmotic stress (Ruiz-Sola et al., 2014). Another group of stress-induced genes includes genes involved in phenylpropanoid biosynthesis pathways. Stronger expression of these genes might synergistically contribute to higher adaptive responses in the tolerant barley.

#### Genes Involved in Osmotic Homeostasis and Accumulation of Compatible Solutes

Plant cells up-regulate the accumulation of compatible solutes (also named osmoprotectants or osmolytes) as specific small organic molecules either by import or *de novo* synthesis to cope with water loss. Downstream of the signaling cascade, TFs are activated by MAPKs, and then trigger the expression of genes that participate in osmoregulation such as *HSPs, LEA*, glycinebetaine (*betA*), and proline (*P5CS*), thereby promoting salt adaptation (Kishor and Sreenivasulu, 2014; Mansour and Salama, 2020). The upregulation of *HvLEA18*, *AvDhn7*, and *HvDhn4* genes belong to LEA proteins in both tolerant and sensitive barleys and *HvHVA22* only in the tolerant genotype under salt stress. These findings may indicate that these proteins are associated with tolerance to salt stress in barley through the membrane and nucleic acid stabilization and chaperon-mediated protection of dehydration-sensitive proteins by forming complexes (Jia et al., 2017). In finger millet, it was found that dehydrins were linked with salt stress tolerance (Rahman et al., 2014), which corroborates our findings. LEA proteins help plants to withstand desiccation and oxidative stress resulting from salt stress. Another protective role of LEA proteins against salt-induced damage is the ability to scavenge ROS (Zheng et al., 2019). Another interesting finding of this study is that those genes (*HvP5CS1* and *HvP5CSB*) that are responsible for the proline accumulation in the leaves were common between the two sub-species. These results are interpreted in support of the concept that they did not play an explicit role in the adaptation response of high salt tolerant grass species (Arabbeigi et al., 2019). There is some controversy about the role of proline in salt tolerance in the literature. But its positive effects on salt adaptation through balancing the osmotic pressure in the cytosol and other intracellular compartments are evident at the low and moderate salt stress or the initial stage of salt exposure.

The up-regulation of two HSPs, in both barleys (wild and Mona cv) enhances membrane stability and detoxify ROS (Haq et al., 2019). Studies have shown that HSP20 genes play a role in growth, development, and stress tolerance in plants. In Tibetan wild barley several HSPs were up-regulated significantly such as HSP17/70 under salt stress (Shen et al., 2018). Guo et al. (2020) have shown that OsHSP20 was induced by heat and high salt stresses in rice and hence may involve in improving tolerance to salt and heat stresses.

In the current study, the *HvSWEET2A* gene was differentially expressed in wild barley. SUGARS WILL EVENTUALLY BE EXPORTED TRANSPORTER (SWEET) gene family is a sugar transporter that is associated with tolerance to oxidative and osmotic stress (Gautam et al., 2019). In wheat, the results provided insights into the role of *TaSWEETs* in abiotic stresses, which may further apply in planning strategies to develop high-yielding wheat tolerant cultivars (Gautam et al., 2019). SWEET family members are found in Arabidopsis in various parts of the cell, including plasma membrane and vacuole membrane. Increased expression of the AtSWEET10 gene in waterlogging stress in Arabidopsis and increased expression of the *BoSWEET* gene in chilling stress in *Brassica oleracea* result in enhanced stress tolerance (Jeena et al., 2019). The expression of the betaine aldehyde dehydrogenase (*HvBADH1*) gene was specifically increased in the tolerant genotype. This enzyme involves in the glycine-betaine biosynthesis which is an effective compatible solute that contributes to maintaining membrane fluidity and protecting the biological structure of the organisms under water and salt stress (Mansour and Ali, 2017). In another study, this gene was induced in the barley leaves under stress and resulted in a high level of glycine-betaine accumulation (Hattori et al., 2009). Up-regulation of HSP chaperones promotes an essential cellular function as they help co-or post-translational protein folding and inhibit protein denaturation or repair it by folding denatured polypeptides. Future studies need to focus on the function and synthesis pathways of genes that regulate an effective collaboration and networks among and within moderate water deficit through osmoregulation, alleviate ion toxicity, scavenging free radicals, and supporting cell and cell membranes.

## Conclusion

Functional analysis of differential transcriptomic data between normal and salt-stress conditions can substantially contribute to the understanding of the mechanisms underlying the inheritance of a complex trait such as salt tolerance in plants. We compared the DEGs between two genotypes/subspecies (wild and cultivated *H. vulgar*) differing in salt tolerance and identified key tolerance-related genes in barley. Indeed, a much stronger transcriptomic response of wild genotype (tolerant) than sensitive ‘Mona’ cultivar as a result of salt stress displays differences in adapting genetic and epigenetic strategies to cope with adverse events associated with salt stress. Perception and signal transmission including plant hormone signal transduction, MAPK signaling pathways, calcium signaling pathways are among the most important pathway components that have been associated with salt tolerance. Our findings of DEGs and pathways will provide more insight into how barley plants can integrate signals and regulatory networks into appropriate adaptation strategies that improve salt tolerance (see Figure 7). The transcriptome dynamics of wild barley in response to salt stress could be exploited, as a bridge from natural habitats, to improve cultivated barley and other cereal crops.

## Data Availability Statement

The original contributions presented in the study are publicly available. This data can be found here: The transcriptome raw data of the present research have been submitted at SRA (Sequence Read Achieve) of NCBI with the accession numbers of SRR16989370, SRR16989367, SRR16989364, SRR16989371, SRR16989366, SRR16989369, SRR16989365, and SRR16989368.

## Author Contributions

AA and MR contributed to the conceptualization and methodology. NG contributed to the experimentation, data analysis, and first draft preparation. AA and RR contributed to the software and validation. AA, MR, and RR contributed to the supervision and manuscript editing. All authors have read and agreed to the published version of the manuscript.

## Conflict of Interest

The authors declare that the research was conducted in the absence of any commercial or financial relationships that could be construed as a potential conflict of interest.

## Publisher’s Note

All claims expressed in this article are solely those of the authors and do not necessarily represent those of their affiliated organizations, or those of the publisher, the editors and the reviewers. Any product that may be evaluated in this article, or claim that may be made by its manufacturer, is not guaranteed or endorsed by the publisher.

## Footnotes

1
http://www.bioinformatics.babraham.ac.uk/projects/fastqc/

2
http://bioinformatics.psb.ugent.be/webtools/Venn/

3
https://biit.cs.ut.ee/gprofiler/gost

4
http://www.pantherdb.org/

5
https://david.ncifcrf.gov/

6
https://www.genome.jp/kegg/kaas/

7
http://primer3.ut.ee/
