# Supplementary material for: Physiological and Transcriptome Indicators of Salt Tolerance in Wild and Cultivated Barley
Source: Front Plant Sci. 2022 Apr 14;13:819282. doi: 10.3389/fpls.2022.819282 (PMC9047362; doi:10.3389/fpls.2022.819282)
Supplement: Supplementary file 1 [file Data_Sheet_1.PDF]

Supplementary Table 1. Primers sequences of the candidate genes used for qRT-PCR reactions.

Gene ID (Contig)	Gene symbol	Gene function	Sequence
HORVU7Hr1G042800	*HvHKT24*	Potassium transporter	F 5'- TGTTTTCTCTCGCGTTCCTT -3'<br>R 5'- GCCACACGCACATCATTATC -3'
HORVU6Hr1G072740	*HvCaSR*	Calcium sensing receptor	F 5'-GGCAGTGTGGACAACTGAAA -3'<br>R 5'- GCGTTTTCTTCTGAGGATGC -3'
HORVU5Hr1G043600	*HvCCDA*	Cytochrome c biogenesis protein family	F 5'-CAGCGATTATGACCCTCGAT-3'<br>R 5'- GAGGCTGCAATGAGAAGAGG -3'
HORVU6Hr1G084010	*AvDhn7*	Dehydrin 7	F 5'- AGGGCATCATGGACAAAATC -3'<br>R 5'- GCTCACTTCATTTCGGGAAG -3'
HORVU2Hr1G077080	*HvCLC-c*	Chloride channel protein CLC-c	F 5'- TCGATCTCCATCCAATCACA -3'<br>R 5'- CTGGTGAGAATCCCAACGAT -3'
HORVU1Hr1G074960	*HvbZIPx*	bZIP domain-containing transcription factor	F 5'- AGTCCGCAAGCTCTGTTGTT -3'<br>R 5'- GAGCCACAATGACAGCAAAA -3'
HORVU3Hr1G007500	*AvHSP20*	HSP20 family protein	F 5'-CAGACAACAACCTACCTACG-3'<br>R 5'-GGTGTCGAACGGGTCAG-3'
Reference gene	*β-tubulin*	Constitutive proteins of microtubules	F 5'- GTCCACCCACTCCCTCCTTG -3'<br>R 5'- CGGCGGCAGATGTCATAGATG -3'

Supplementary Table 2. Functional annotations of some of the differentially-expressed (up and down regulated) genes in wild (tolerant) and cultivated (sensitive) genotypes of barley grown under control and salt stress conditions

Gene ID	Gene function	Gene symbol	Tolerant (Log2FC)	*P*-value	Sensitive (Log2FC)	*P*-value
**HORVU1Hr1G081310**	Snf1-related protein kinase 1	*HvSnRK1 alpha2*	2.007	0.000006	0.51	0.204931
**HORVU4Hr1G056610**	Snf1-related protein kinase 1	*HvSnRK1 alpha3*	3.035	0.0000007	1.08	0.025015
**HORVU1Hr1G000310**	Snf1-related protein kinase 1	*HvSnRK1 beta3*	1.6	0.0014	0.53	0.196922
**HORVU4Hr1G022630**	Snf1-related protein kinase 1	*HvSnRK3*	6	0.000000012	0.32	0.708804
**HORVU7Hr1G027810**	Calcium-binding EF-hand family protein	*HvCML48*	4.6	0.0000057	0.94	0.69331
**HORVU3Hr1G109230**	Calcium-binding EF-hand family protein	*HvCML31*	6.29	0.000031	0.94	1
**HORVU6Hr1G091790**	Calmodulin-binding receptor-like cytoplasmic kinase 1	*HvCaMBP1*	4.31	0.0000029	0.44	0.78
**HORVU6Hr1G072740**	Calcium sensing receptor	*HvCaSR*	-3.91	0.0000000001	-0.811	0.016
**HORVU2Hr1G101040**	Calcium-transporting ATPase	*ATP2* (syn. *PMCA*)	-1.64	0.0018	-0.29	0.483
**HORVU6Hr1G085890**	ATP-binding cassette (ABC) transporter	*HvABCF3*	1.78	0.00012	0.52	0.15
**HORVU4Hr1G018800**	ATP-binding cassette (ABC) transporters	-	0.0	1	1.78	0.000938
**HORVU7Hr1G042800**	Potassium transporter	*HvKT24*	4.98	0.00000005	-0.14	0.863
**HORVU7Hr1G109770**	Vacuolar proton-ATPase (V-ATPase)	*HvVA68*	2.05	0.0000012	0.25	0.476
**HORVU4Hr1G062880**	V-type proton ATPase subunit A	*HvVPH1* (syn. *Hv ATP6V1A*)	2.04	0.00000044	0.48	0.16
**HORVU6Hr1G019930**	Protein ABC transporter 1	*HvABC1*	2.05	0.00034	-0.22	0.733
**HORVU2Hr1G077080**	Chloride channel protein CLC-c	*HvCLC-c*	1.50	0.00038	1.04	0.00445
**HORVU3Hr1G068140**	Sulfate transporter 3.5	*HvSultr3;5*	8	0.000001	1.68	1
**HORVU2Hr1G100440**	High affinity K^+ transporter	*HvHKT1;2*	2.47	0.078	0.0	1
**HORVU5Hr1G094840**	Cytosolic FE-S cluster assembly factor (family: nitrate, formate, iron dehydrogenase)	*HvNAR1*	3.58	0.000000079	0.65	0.173
**HORVU6Hr1G029520**	Bidirectional sugar transporter	*HvSWEET2A*	2.09	0.0000055	-0.51	0.348

**HORVU3Hr1G082230**	Probable magnesium transporter	-	2.81	0.00000035	-0.37	0.496
**HORVU2Hr1G127500**	Probable metal ion transporter	-	2.04	0.000043	0.71	0.240
**HORVU4Hr1G033760**	Sodium/hydrogen exchanger 2	*AvNHX1*	1.50		1.21	0.017
**HORVU2Hr1G102840**	Vacuolar cation/proton exchanger 2B	*HvCAX2B*	4.02	0.0000003	-0.73	0.692
**HORVU3Hr1G049060**	Vacuolar cation/proton exchanger	*HvCAX1A*	1.5	0.0016	0.45	0.172
**HORVU3Hr1G058300**	AKT1 potassium channel	*HvAKT1*	3.72	0.00000066	1.23	0.101
**HORVU7Hr1G040990**	SKOR potassium channel	*HvSKOR*	1.71	0.010	2.1	0.00043
**HORVU6Hr1G070120**	Thioredoxin reductase	*HvTrxR1*	2.68	0.000013	0.41	0.139
**HORVU6Hr1G091330**	Thioredoxin-like 3-2, chloroplastic	*HvTrxL3-2*	2.64	0.000000026	1.07	0.0311
**HORVU5Hr1G117910**	Ferredoxin-3	*HvFdx3*	3.10	0.00000024	0.59	0.203
**HORVU3Hr1G084210**	Ferredoxin 3	*HvFdx3*	6.38	0.00000000042	3.19	0.5
**HORVU4Hr1G043910**	Protein disulfide-isomerase	*HvPDI*	2.26	0.000000059	1	0.00077
**HORVU2Hr1G080970**	Betaine aldehyde dehydrogenase 1	*HvBADH1*	3.06	0.000000043	1.10	0.00015
**HORVU2Hr1G004720**	Glycosyltransferase	-	11.38	0	2.96	0.065
**HORVU2Hr1G019680**	Glycosyltransferase	-	8.75	0	0.65	1
**HORVU7Hr1G038510**	Glycosyltransferase	-	5.72	0.0000000052	-0.05	1
**HORVU5Hr1G085310**	Glycosyltransferase	-	3.88	0.000000384	1.04	0.02020
**HORVU5Hr1G068330**	ABA 8'-hydroxylase 2	*HvCYP707A4*	5.15	0.00000000007	-1.28	0.00071
**HORVU2Hr1G021110**	Cu-Zn superoxide dismutase family	*HvSOD1*	1.81	0.000039	0.90	0.0088
**HORVU7Hr1G121700**	Catalase	*HvCAT1*	4.22	0	0.3	0.3941
**HORVU3Hr1G074940**		*HvPOD*	4.1	0.0023	-2.27	0.049
**HORVU3Hr1G074960**	peroxidase	*HvPOD*	8.1	0.00000000007	-1.3	0.62
**HORVU1Hr1G013950**	bZIP domain-containing tra cription factor	*HvbZIPx*	2.23	0.0000000011	1.19	0.0009
**HORVU1Hr1G060810**	Gibberellin hormone receptor	*HvGID1c*	2.11	0.001	1.32	0.003
**HORVU3Hr1G018860**	3-Epi-6-deoxocathasterone 23-monooxygenase	*HvCYP90D1*	2.97	0.0000000003	-0.12	0.82

**HORVU7Hr1G003170**	Lipoxygenase	-	3.7	0.000089	6.5	0.00000049
**HORVU4Hr1G005920**	Lipoxygenase	*HvLoxB*	2.69	0.00000007	0.63	0.015
**HORVU4Hr1G057210**	ascorbate peroxidase	*HvAPX*	1	0.13	1.55	0.0004
**HORVU7Hr1G003170**	Late embryogenesis abundant protein	*HvLEA18*	6.56	0.000089	3.73	0.00000408
**HORVU6Hr1G084010**	Dehydrin 7	*AvDhn7*	4.24	0.000018	7.28	0
**HORVU6Hr1G084070**	Dehydrin 4	*HvDhn4*	5.63	0.00043	7.37	0
**HORVU6Hr1G012260**	HVA22-like protein	*HvHVA22*	1.51	0.00911	0.45	0.39
**HORVU1Hr1G072780**	Delta-1-pyrroline-5-carboxylate synthase	*HvP5CS1*	2.35	0.00000056	2.16	0.0021
**HORVU3Hr1G085760**	Delta-1-pyrroline-5-carboxylate synthase	*HvP5CSB*	3.44	0.0039	4.71	0.000000085
**HORVU1Hr1G080320**	Aldehyde dehydrogenase family 12 member A1	*HvALDH12A1*	3.80	0.000000001	1.43	0.0025
**HORVU0Hr1G020420**	15.7 kDa heat shock protein	*AvHSP15.7*	7.28	0	3.88	0.00108
**HORVU3Hr1G007500**	HSP20 family protein	*AvHSP20*	5.38	0	2.84	0.0000002
**HORVU5Hr1G036590**	AP2/ERF-ERF	*HvAp2/ERF*	4.30	0.0000000027	-0.18	1
**HORVU5Hr1G062940**	AP2/ERF-ERF	*HvAp2/ERF*	3.96	0	0.28	0.49
**HORVU2Hr1G071270**	AP2/ERF-ERF	*HvAp2/ERF*	7.43	0.00000000004	-0.42	0.999
**HORVU0Hr1G013950**	bZIP	*HvbZIP16*	5.83	0	3.08	0.12
**HORVU5Hr1G106120**	bZIP	*HvbZIP63*	2.75	0.000001	-0.57	0.29
**HORVU2Hr1G119610**	MYB	*HvMYB*	3.01	0.00000002	0.94	0.011
**HORVU1Hr1G063740**	NAC	*HvNAC56*	4.80	0.00000000002	3.10	0.0000006
**HORVU3Hr1G090920**	NAC	*HvNACx*	2.24	0.0000007	1.74	0.000002
**HORVU2Hr1G001780**	WRKY transcription factor 75	*HvWRKY75*	4.91	0.0000000078	0.59	0.78
**HORVU7Hr1G094690**	bHLH	*HvbHLH*	4.2	0.000000007	-4.05	0.0004
**HORVU7Hr1G026940**	AP2/ERF-ERF	*HvAp2/ERF*	1.78	0.013	1.73	0.0022
**HORVU6Hr1G008930**	RLK-Pelle_LRR-XII-1	-	-5.10	0.00000005	-0.81	0.32
**HORVU5Hr1G119270**	RLK-Pelle_WAK	-	8.01	0	0.7	1

**HORVU4Hr1G045160**	CAMK2;calcium/calmodulin-dependent protein kinase (CaM kinase) II	-	4.7	0.0000006	0.06	1
**HORVU2Hr1G110230**	Stress-activated protein kinase 1	*HvSAPK1*	1.20	0.0006	2.94	0
**HORVU4Hr1G057200**	Mitogen-activated protein kinase (MAPK)	-	2.2	0.000015	0.36	0.4
**HORVU4Hr1G006660**	MAP3K	-	2.27	0.0023	0.24	0.77
**HORVU1Hr1G078860**	MAP3K	-	4.31	0.017	3.02	0.5
**HORVU4Hr1G060940**	COI1	*HvCOI1*	1.61	0.0049	2.2	0.00037
**HORVU2Hr1G070880**	JAZ	*HVJAZ*	5.73	0.000000008	-0.014	1
**HORVU0Hr1G016330**	PAL	*HvPAL*	1.9	0.000015	-.058	0.089
**HORVU1Hr1G090360**	PP2C	*HvPP2C*	4.34	0	3.6	0.066
**HORVU4Hr1G002330**	Na^+/Ca^{2+} exchanger	*HvNCKX*	2.9	0.022	1.03^{ns}	0.69
**HORVU2Hr1G094160**	beta-carotene 3-hydroxylase	*HvCHYB*	2.9	0.00002	-0.32	0.5

- a hitherto un-named gene
