# Supplementary material for: Physiological and Transcriptome Indicators of Salt Tolerance in Wild and Cultivated Barley
Source: Front Plant Sci. 2022 Apr 14;13:819282. doi: 10.3389/fpls.2022.819282 (PMC9047362; doi:10.3389/fpls.2022.819282)
Supplement: Supplementary file 2 [file Data_Sheet_2.pdf]

Supplementary Figures 1-5

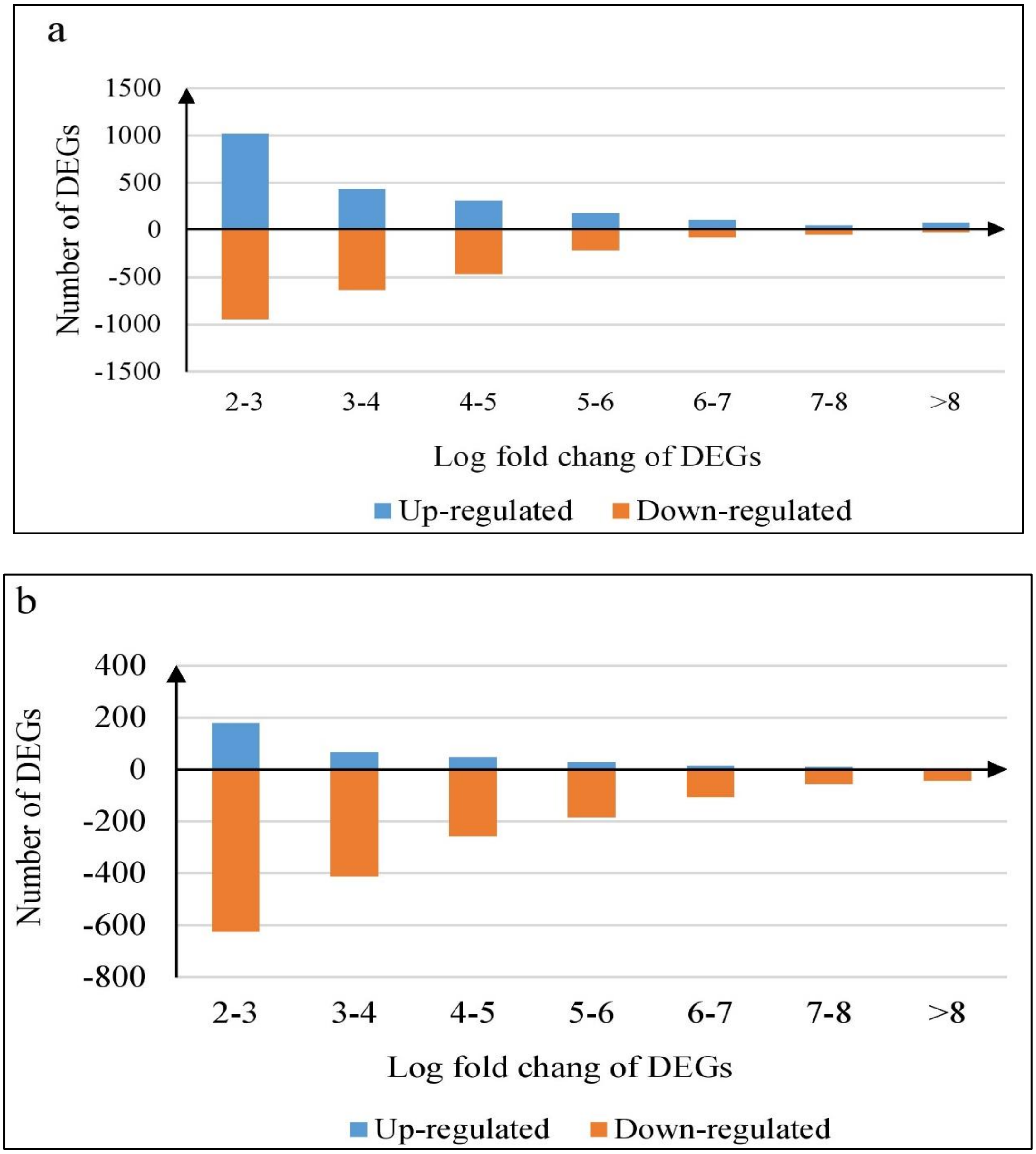

Supplementary Figure 1. Histogram of the fold change in expression of the DEGs (Log2 FC) of two barley subspecies; (a) wild salt-tolerant genotype and (b) 'Mona' salt-sensitive cultivar

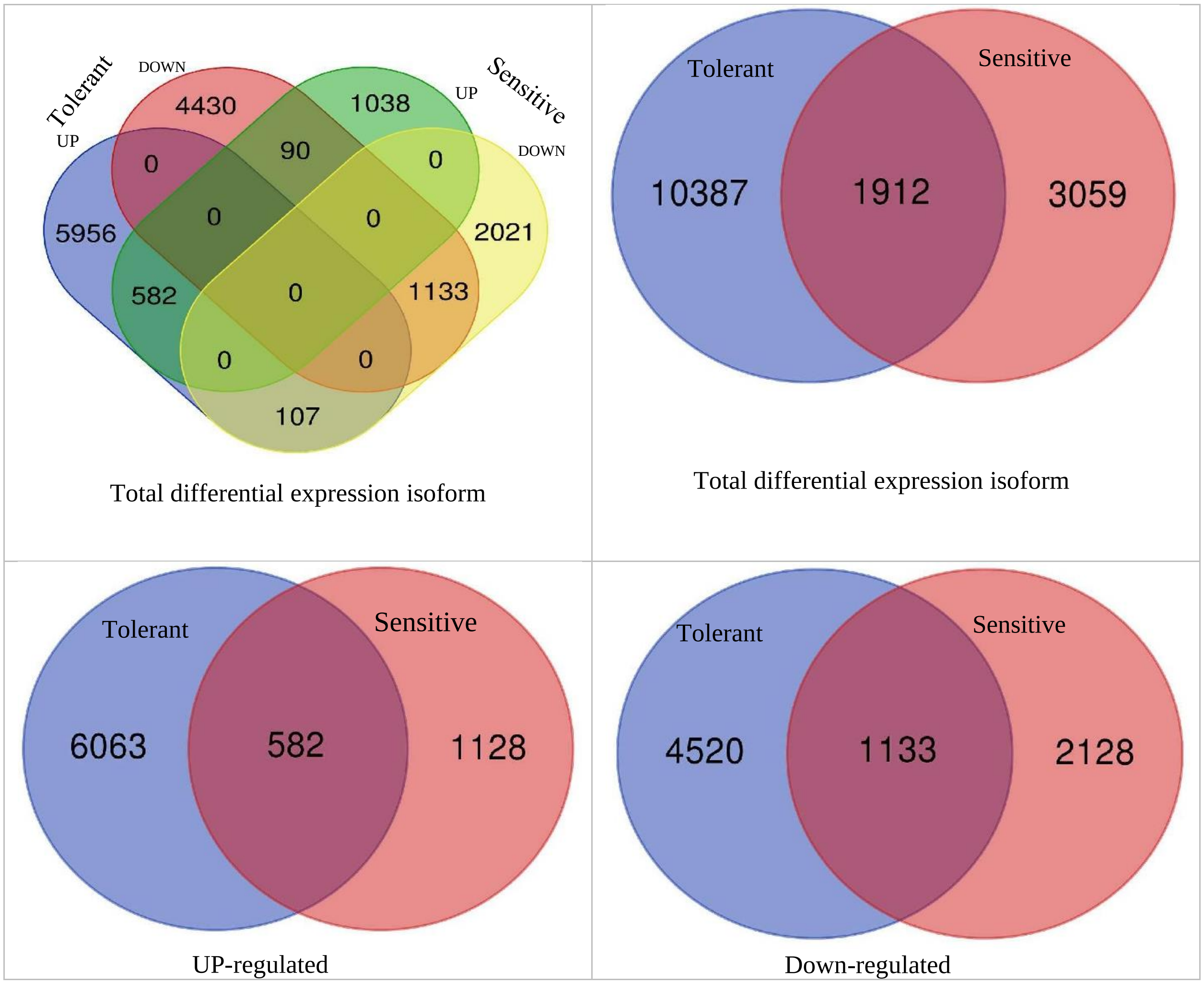

Supplementary Figure 2. Venn diagrams showing the number of distinct and common salt-responsive isoforms (DEIs) found in two barley subspecies (wild salt-tolerant genotype and 'Mona' salt-sensitive cultivar).

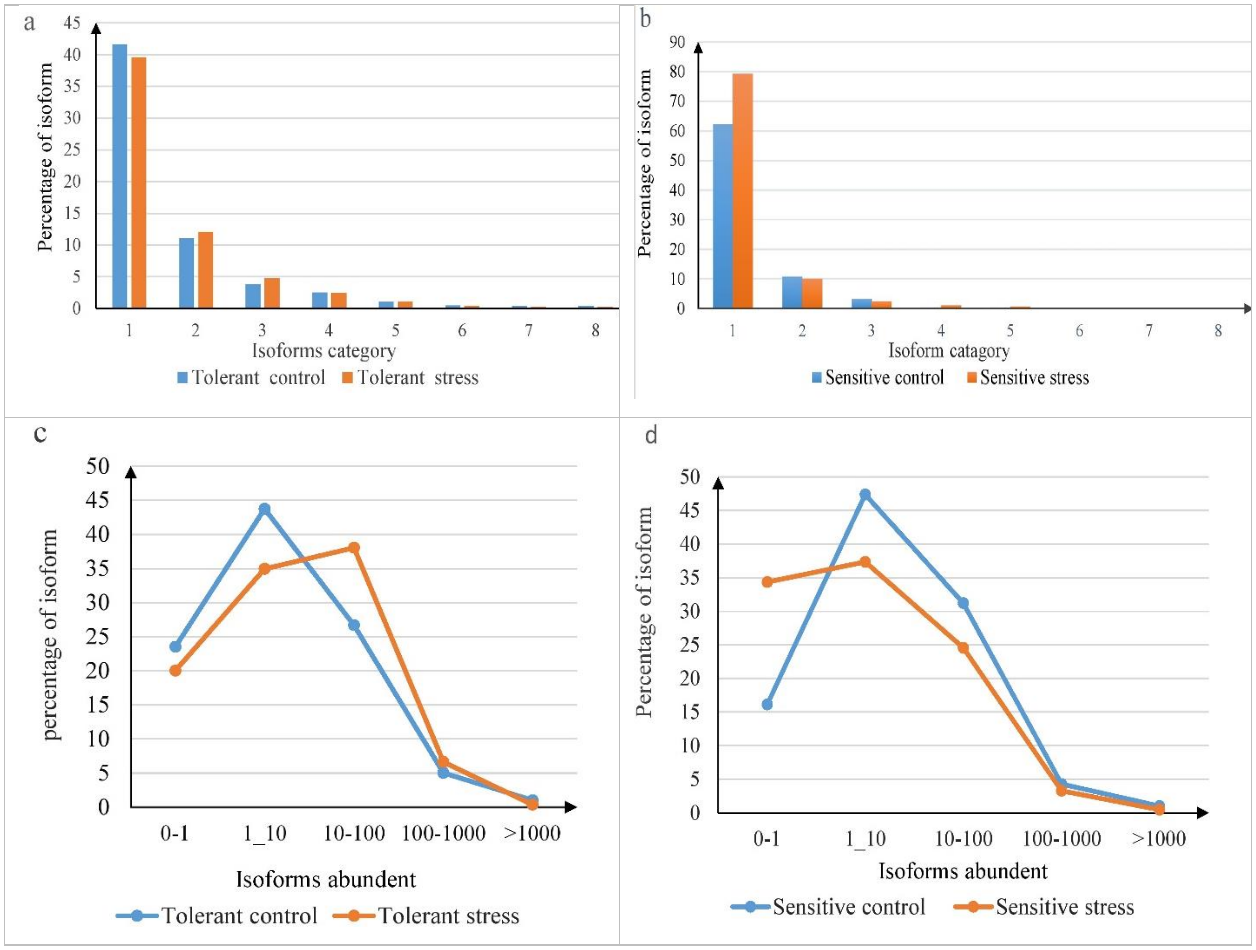

Supplementary Figure 3. Frequency distribution of number of isoforms per gene (a and b) and their abundance (c and d) in two *H. vulgare* subspecies (salt tolerant and sensitive). Frequency of the top 8 groups for (a) salt tolerant genotype and (b) salt sensitive cultivar. The isoform abundance distribution for (c) salt tolerant genotype and (d) salt sensitive cultivar.

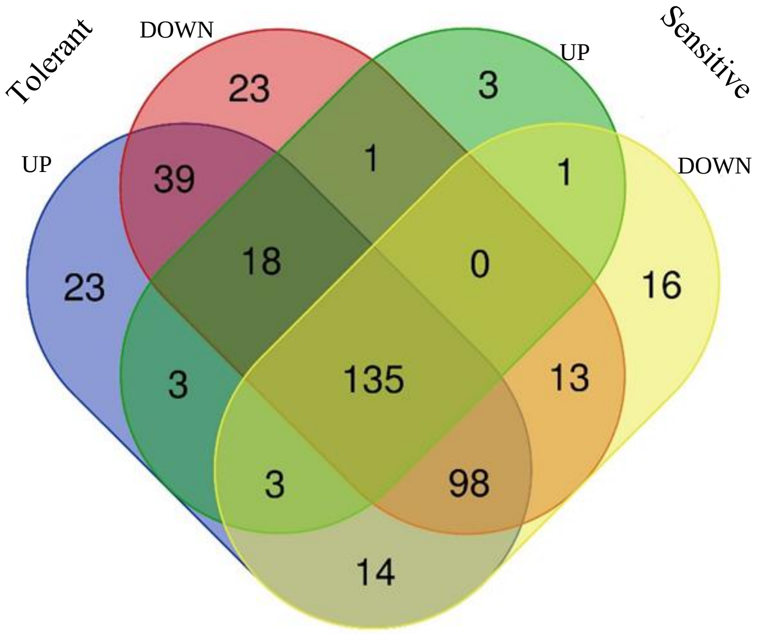

Supplementary Figure 4. Venn diagrams showing the numbers and overlap of up- and down-regulated KEEG pathways found in two barley subspecies (wild salt-tolerant genotype and 'Mona' salt-sensitive cultivar).

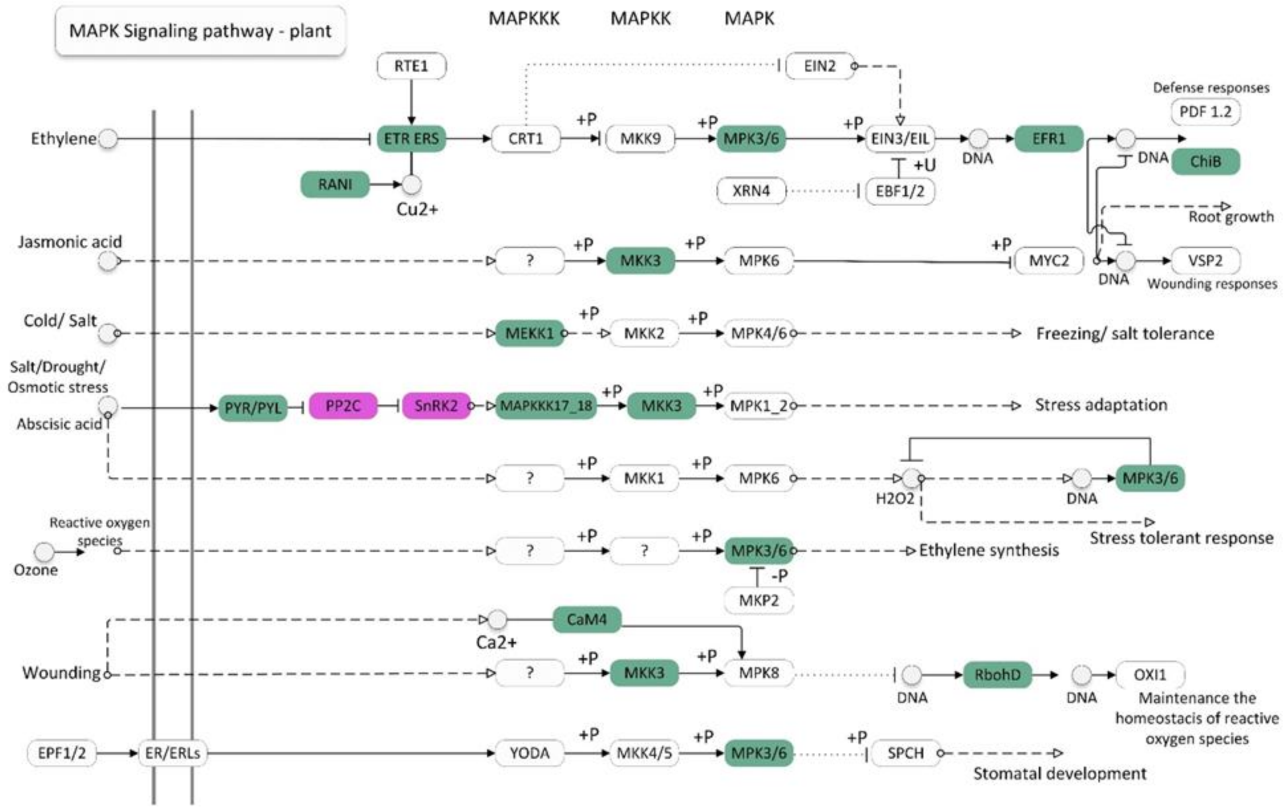

Supplementary Figure 5. MAPK signal transduction detected in response to salt stress conditions (300 mM NaCl) in barley. This figure provides only a KEGG pathway for up-regulated genes in the salt-tolerant genotype and salt-sensitive cultivar. Green color boxes represent the common differentially expressed genes (DEGs) between salt-tolerant and salt-sensitive barleys and purple color boxes represent the specifically DEGs in salt-tolerant genotype. EC number of the encoded enzyme is shown in each box.
